# Foot exercise plus education versus wait and see for the treatment of plantar heel pain (FEET trial): a protocol for a feasibility study

**DOI:** 10.1186/s13047-020-00384-1

**Published:** 2020-05-08

**Authors:** Melinda M. Franettovich Smith, Natalie J. Collins, Rebecca Mellor, Alison Grimaldi, James Elliott, Mark Hoggarth, Kenneth A. Weber II, Bill Vicenzino

**Affiliations:** 1 School of Health and Rehabilitation Sciences, The University of Queensland, Brisbane, Queensland 4072 Australia; 2 La Trobe Sport and Exercise Medicine Research Centre, School of Allied Health, Human Services and Sport, College of Science, Health and Engineering, La Trobe University, Melbourne, 3086 Australia; 3 PhysioTec Physiotherapy, Brisbane, Queensland 4121 Australia; 4 Faculty of Medicine and Health and The Kolling Research Institute, The University of Sydney, Sydney, New South Wales 2006 Australia; 5 Department of Physical Therapy and Human Movement Sciences, Northwestern University, Chicago, IL USA; 6 Systems Neuroscience and Pain Lab, Division of Pain Medicine, Department of Anesthesiology, Perioperative and Pain Medicine, Stanford University School of Medicine, Palo Alto, California USA

**Keywords:** Plantar fasciitis, Exercise therapy, Rehabilitation, Intrinsic foot muscles, Physiotherapy, Randomised control trial

## Abstract

**Background:**

Plantar heel pain (PHP) is present in a wide range of individuals and creates significant burden to quality of life and participation in physical activity. The high recurrence rates and persistence of PHP suggests current management options may not address all potentially modifiable factors associated with the condition. Reports of intrinsic foot muscle (IFM) atrophy in individuals with PHP, together with biomechanical evidence of their important contribution to optimal foot function, suggests that an intervention focused on IFM training may be beneficial in managing PHP. We will test the feasibility of a prospective, assessor-blinded, parallel-group, randomised clinical trial that compares foot exercise plus education to brief advice in individuals with PHP.

**Methods:**

Twenty participants with PHP will be randomly allocated to one of two groups for a 12-week intervention period: (i) foot exercise plus education, or (ii) brief advice. The foot exercise plus education group will attend eight sessions with a physiotherapist and receive detailed education on self-management strategies as well as a progressive exercise program for the IFMs. The brief advice group will attend one session with a physiotherapist and receive brief information about self-management strategies and reassurance. Outcome measures will be obtained at baseline and the primary end-point of 12 weeks. Primary outcomes will be the feasibility of conducting a full-scale randomised clinical trial (RCT), and the credibility and acceptability of the foot exercise plus education intervention. Secondary outcomes will explore treatment effects, which will consist of pain, physical function, physical activity level, pain self-efficacy, perceived treatment effect, magnetic resonance and ultrasound image measurement of IFM morphology, ultrasound imaging measurement of plantar fascia thickness, IFM motor performance, foot posture, foot mobility, ankle dorsiflexion range of motion, toe flexor and plantar flexor strength/endurance.

**Discussion:**

To reduce the burden of PHP on individuals and society, there is a need to establish effective treatments that are feasible and accepted by patients and health professionals. This trial will be the first to evaluate the feasibility of conducting a full-scale RCT, as well as the credibility, acceptability, and treatment effects, of education and foot exercise for PHP. The findings of this study will inform the development of a full-scale RCT.

**Trial registration:**

The trial protocol was prospectively registered with the Australia and New Zealand Clinical Trial Registry (ACTRN12619000987167) on 11th July 2019.

## Background

Plantar heel pain (PHP) is a common musculoskeletal disorder characterised by pain at the plantar aspect of the medial heel, most noticeable on weight bearing after a period of inactivity but also worse following prolonged weight bearing [1]. PHP affects a wide spectrum of individuals from the sedentary to the physically active/athletic individual. Prevalence rates vary between 3 and 18% among different populations [2–8]. Low physical activity and higher rates of anxiety, depression and stress are reported by individuals with PHP, highlighting the significant burden of the condition beyond pain [2, 9]. Despite the common description of PHP as a ‘self-limiting’ disorder, a longitudinal study with an average follow-up of 10 years reported that 53% of individuals experienced relapse of symptoms in that time, and 46% had persistent symptoms [10]. This suggests that current management options do not adequately address symptoms and impairments associated with the condition, nor address potential contributors to ongoing symptoms.

During walking and running the foot dissipates, recycles and produces mechanical energy [11]. This involves contributions from both the passive (i.e. plantar fascia, heel fat pad, ligaments and bone) and active (i.e. intrinsic and extrinsic muscles) structures of the foot. The intrinsic foot muscles (IFMs) have all their anatomical attachments within the foot, as opposed to the extrinsic muscles that originate in the leg. The IFMs have been shown to assist damping of energy associated with foot-ground impact and play a critical role in stiffening the foot for propulsion [12, 13]. The distinguishing contribution of the IFMs (compared to passive structures) is the ability to actively modulate the energetic function of the foot to respond to changing demands (e.g. acceleration/deceleration, surface, and footwear, etc.) [11, 14].

Muscle morphology is a primary determinant of muscle function [15]. Atrophy of the IFMs has been reported in PHP. A previous study that used magnetic resonance imaging (MRI), the gold-standard for evaluation of muscle morphology, reported smaller rearfoot muscle volume in runners with persistent PHP when compared to asymptomatic runners [16]. The IFMs were segmented as a muscle group (not individual muscles) by excluding all non-contratile tissues on each image. IFM volume was then calculated by summing the product of slice thickness and IFM cross-sectional area for each image. Forefoot and rearfoot volumes were determined from the anterior or posterior half of the full image set, respectively, and therefore also represent morphology of a region rather than individuals muscles. Due to the cross-sectional design of previous research, the causal relationship between IFM morphology and PHP is unknown. It is possible that IFM atrophy contributes to the development of PHP. For example, atrophy of these muscles may compromise the energetic function of the foot with implications (e.g. modified loading) for passive structures such as the plantar fascia, heel fat pad and calcaneus. It is also plausible that IFM atrophy is a consequence of PHP. For example, IFM atrophy may occur due to the presence of pain and/or altered foot and physical function. The significant contribution of the IFMs to the energetic function of the foot suggests that IFM atrophy is important to address in the management of PHP.

Three studies have evaluated interventions for PHP that have included IFM training [17–19]. Two of these studies [18, 19] have investigated 4 weeks of multimodal interventions where IFM exercise was combined with manual therapy, electrotherapy, stretching and/or medication. In these studies, as IFM exercise was a component of a combined package of interventions, and was included in the intervention for both comparator groups, the effect of IFM training is difficult to determine. The other study [17] investigated the addition of IFM exercise to calf stretching over 8 weeks and reported no difference when compared to calf stretching alone. In that study, there were several limitations that may have influenced the effectiveness of the IFM exercise programme. For example, there were only two exercises that targeted the IFMs (toe curl exercise and short foot exercise) that were performed under supervision of a physiotherapist for 8 weeks (16 sessions in total). The toe curl exercises were performed for 3 sets of 15 repetitions with graduated resistance of 1 and 2 kg weights and the short foot exercise for 3 one-minute isometric holds in single limb stance. The exercise programme was likely insufficient for hypertrophy/strength gains [20], but it remains unknown, as there was no measurement of IFM structure and/or strength. Two other studies in healthy populations, have reported increased IFM size (10–21%) and strength (35–106%) with a programme that involved more than two foot exercises, at higher intensities and larger volumes [21, 22]. IFM training with sufficient volume, intensity and progression may produce more beneficial results in PHP compared to previous trials.

The primary aim of this study is to investigate the feasibility of conducting a full-scale randomised clinical trial (RCT), as well as the credibility and acceptability of a foot exercise plus education intervention in individuals with PHP. The secondary aim is to investigate treatment response (effect sizes) using a comprehensive set of measurements which consists of pain, physical function, physical activity level, pain self-efficacy, perceived treatment effect, MRI and ultrasound image measurement of IFM morphology, ultrasound imaging measurement of plantar fascia thickness, IFM motor performance, foot posture, foot mobility, ankle dorsiflexion range of motion, toe flexor and plantar flexor strength/endurance.

## Methods

### Design

**Fig. 1:**
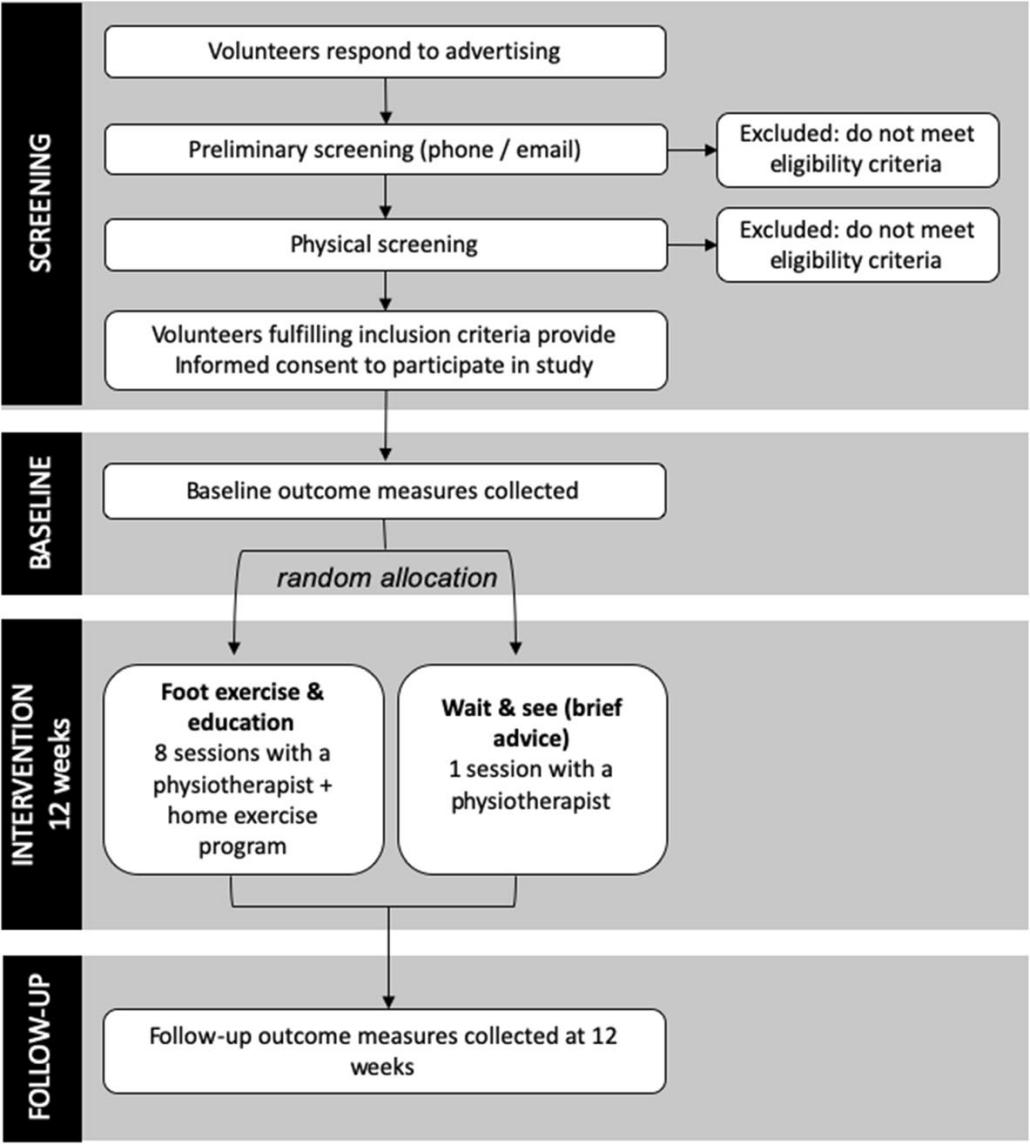
Study design

This protocol describes a feasibility study of a prospective, assessor-blinded, randomised clinical trial with two parallel groups and a 12-week intervention period (Fig. 1). Participants will be randomised to each group using a 1:1 ratio (see ‘Allocation’ for more detail regarding randomisation). The primary end point is 12 weeks. The trial will be conducted in physiotherapy clinics in Brisbane, Australia and The University of Queensland. This study protocol follows the Standard Protocol Items: Recommendations for Interventional Trials (SPIRIT) guidelines [23]. The trial was prospectively registered with the Australia and New Zealand Clinical Trial Registry on 11th July 2019 (ACTRN12619000987167; http://www.ANZCTR.org.au/ACTRN12619000987167.aspx).

### Ethics approval

Ethical approval was obtained from The University of Queensland’s Human Research Ethics Committee (#2019000772). Prior to enrolment in the trial, all participants will provide informed written consent.

### Participant recruitment

Potential participants will be recruited from the Brisbane community using social media (Facebook) and public advertisements (University and community noticeboards).

### Eligibility criteria

A three-stage screening process (online survey, telephone interview, physical screening) will be used to assess eligibility of volunteers who respond to advertisements. The advertisement will direct volunteers to an online survey link (Google forms) or to contact the chief investigator (email, phone). The online survey link will assess preliminary eligibility criteria (e.g. age, pain characteristics and concomitant conditions, see Additional File 1). For volunteers who directly contact the chief investigator, the preliminary eligibility criteria will be covered within the telephone interview. Telephone interviews and physical screenings will be conducted by the chief investigator (see Additional File 2), a registered physiotherapist with 15 years of experience. The telephone screening will assess eligibility criteria in more detail and describe the purpose of the study and participant involvement in more detail. Volunteers who fulfil all eligibility criteria, and are able to commit to participation in the trial, will be invited to attend a physical screening at The University of Queensland. The physical screening will involve palpation of the plantar fascia and medial calcaneal tubercle and ultrasound image measurement of plantar fascia thickness.

#### Inclusion criteria

Volunteers will be included if they satisfy the following criteria.

1. Over 18 years of age.
2. Report a history of insidious onset pain under the sole of the foot, located at the heel, that has persisted for more than 3 months.
3. Report pain that is most noticeable with initial steps following a period of inactivity, and also worsened with prolonged weight bearing [1].
4. Report worst first step pain during the previous week of greater than 3/10 on a numerical rating scale (0 = no pain; 10 = worse pain imaginable).
5. Report pain on palpation of the medial calcaneal tubercle or proximal fascia.
6. Plantar fascia thickness is 4 mm or greater when measured using ultrasound imaging.

### Exclusion criteria

Volunteers will be excluded if they report a history of the following:

1. Diabetes or an inflammatory systemic disease.
2. Concomitant foot injury or pathology.
3. Previous foot surgery.
4. Pain or an injury of the lower back or lower limb (other than heel pain) in the preceding 6 months that caused them to seek medical assistance or treatment, take medication, miss work or reduce physical activity for at least 1 week.
5. Currently pregnant or breastfeeding.
6. Have received a corticosteroid injection in the foot during the preceding 6 months.
7. Contraindications to undergoing MRI (e.g. metal implants or pacemaker, etc.).

### Informed consent

Volunteers who meet the eligibility criteria will be provided with a participant information and consent form and given the opportunity to ask any questions of the chief investigator. Eligible volunteers who decide to participate will provide written informed consent and a baseline assessment session will be scheduled.

### Allocation

Following baseline assessment, participants will be randomised via concealed allocation to either foot exercise and education or wait and see (brief advice). A computer generated randomization schedule will be used, allocating on a 1:1 ratio in random permuted blocks of 2–6 [24]. A study investigator (RM) who is not involved in recruitment or outcome assessment will manage the randomisation allocation and liaise with the treating physiotherapist regarding group allocation. As treating physiotherapy clinics are located in various suburbs of Brisbane, participants may select the most convenient practice for them to attend. Participants will be informed of group allocation by the treating physiotherapist.

The interventions will be delivered by registered physiotherapists, who regularly treat musculoskeletal conditions, in seven private practices across Brisbane. To ensure consistent implementation of both interventions, treating physiotherapists will attend 2 training sessions of 90-min duration each, 1 week apart, at The University of Queensland prior to commencement of the trial. Trial physiotherapists will also be provided with a trial handbook (detailed documentation including images, instructions and progressions of exercises) and advised to contact the study investigators for any queries that arise during the trial.

### Interventions

#### Foot exercise plus education

Participants allocated to this group will attend eight individual physiotherapy sessions (weeks 1,2,3,4,5,6,8 and 10) during the 12-week intervention period. Physiotherapy sessions will be 30 min duration and will include detailed education relating to the condition and self-management strategies, as well as an exercise program that will be performed both at home and supervised by the physiotherapist during sessions. Participants will be asked to complete a daily logbook (Additional file 3) of exercises that are completed, symptoms, any physical activity that is undertaken, adverse responses and details of any treatments they have outside of the study protocol.

The 12-week foot muscle focussed training programme will consist of two components: exercises performed daily and exercises performed three times a week. Table 1 provides an overview of the exercise programme and Additional file 4 provides the detailed exercise protocol, including specific instructions for each exercise. Daily exercises will consist of four foot and toe movements and two weight bearing exercises. Exercises performed three times a week will consist of four exercises, each with three stages of progression. Progression is provided by varying the number of repetitions and sets, amount of load and rate of loading. Participants will progress through the stages 1 to 3 in an individualized, criterion-based manner. When the indicated repetitions and sets are reached for a stage, progression is made provided that:

(i) the therapist considers that the patient can adequately recruit the IFMs without excessive extrinsic foot muscle contribution (e.g. able to maintain extension of the interphalangeal joints of the toes) or perform local joint motion at the foot and ankle with optimal control (i.e. smooth motion through the intended plane of action without unintended overflow into adjacent planes or joints, such as, pronation or supination of the foot and ankle);
(ii) the participant’s rating of effort of each exercise using Modified Borg Scale is 0–5/10;
(iii) the participant’s pain response to loading is adequate (pain during exercise < 5/10 on a numerical rating scale and no change in first step pain the following day).

**Table 1:**
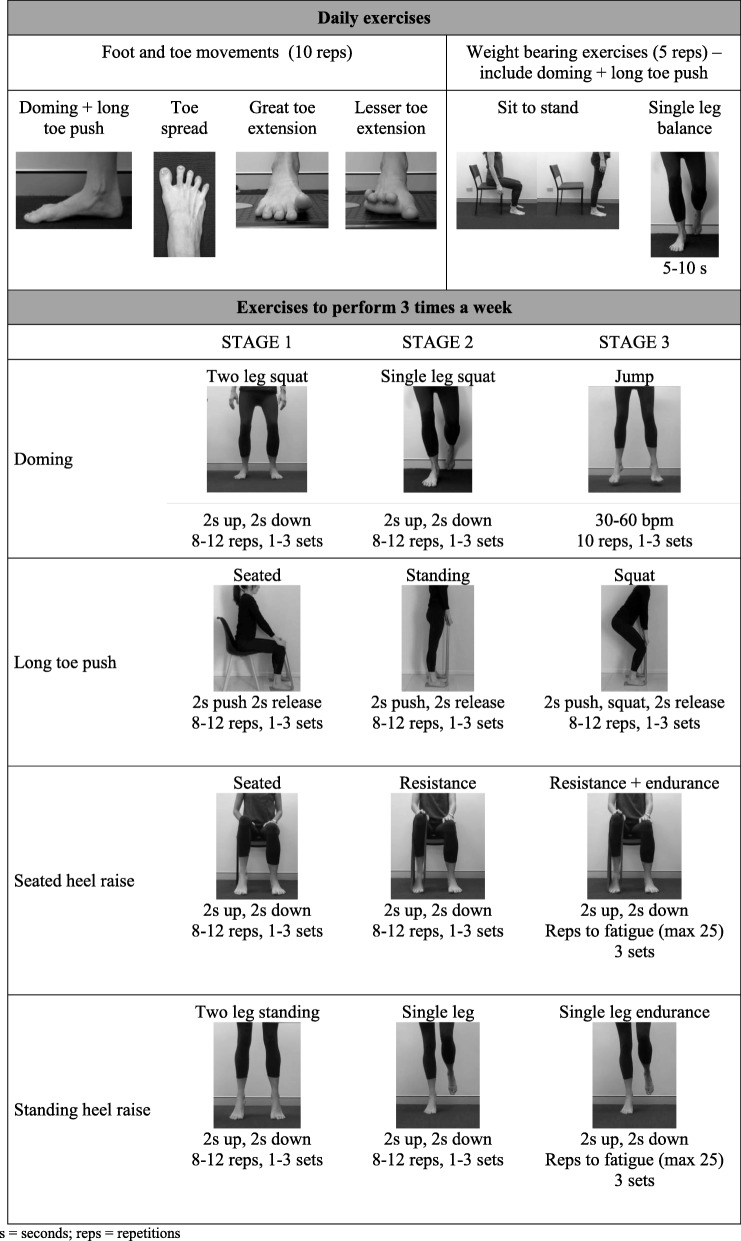
Exercise programme

*s* seconds; *reps* repetitions

The education component will include information about the condition and self-management strategies. Information about the condition will outline that PHP is a description of symptoms, and that there can be several structures involved and common contributing factors. Self-management advice will explain how to monitor load and symptom response and advice regarding footwear, posture and gait. This will be delivered one-on-one by the physiotherapist at the first session and supported by the provision of a handout detailing the information discussed with the physiotherapist (Additional file 5). Review and reinforcement of self-management strategies will be included at each subsequent physiotherapy sessions.

#### Wait and see (brief advice)

Participants allocated to this group will attend one individual (one-on-one) physiotherapy session during the 12-week intervention period (week 1). The session will be 30 min duration and consist of the therapist providing brief information about the condition and advice on how to self-manage the condition (e.g. common contributing factors, modifying daily activities to within acceptable pain levels and gradual progressions in activity within pain limits, etc.). Participants will be reassured that in many people symptoms can resolve over time (12–24 months) and that currently there is no gold standard treatment approach supported by scientific evidence. The physiotherapist will take time to listen to any patient concerns regarding their condition. Information will be supported by provision of a simple handout (Additional file 6). Participants will receive no additional treatment during the 12-week intervention period. Participants will be asked to complete a weekly logbook of symptoms, any problems that are encountered (related to their heel pain) and any treatments outside of the study protocol (Additional file 7).

#### Adverse events

Participants will be encouraged to report any untoward health/medical related events (i.e. adverse events) regardless of likely cause. This will include possible adverse reactions (e.g. increase in heel pain, onset of new pain or injury elsewhere) that they experience during the study to a study investigator (RM) and/or their physiotherapist. If an adverse event is associated with the foot exercise plus education intervention, the physiotherapist will modify the exercise prescription and/or load management advice accordingly until a reduction in symptoms is reported. In the event of a sustained increase in heel pain or aggravation of another area of pain, standard clinical practice principles will apply and provision of any treatments outside of the study protocol (e.g. taping) will be recorded in the physiotherapist’s treatment notes and reported to a study investigator (RM). If symptoms associated with the adverse event are unable to be resolved, the intervention will be ceased and the participant encouraged to remain in the trial to enable follow-up data collection.

### Outcome measures

To ensure sufficient information is obtained to inform the design of a full-scale RCT, a comprehensive set of primary and secondary outcome measures will be recorded (Table 2). Outcome measures will be assessed at baseline and 12-week time-points (at the end of the intervention period). The 12-week time period for measuring outcomes allows an adequate timeframe to observe adaptations, allowing for (a) a 2-week lead in period of familiarisation and movement competency training, (b) an 8-week strengthening programme, (c) graduated progression of exercise, and (d) integration into functional tasks. All outcome measures will be obtained by an assessor who is blinded to group allocation (MMFS).

**Table 2:** Outcome measurements

		STUDY PERIOD		
		Enrolment/ Baseline	Post-allocation	Close-out
**TIMEPOINT**^**a**^		**t**_**0**_	**t**_**1**_	**t**_**2**_
**ENROLMENT:**				
Eligibility screen		X		
Informed consent		X		
Allocation		X		
**INTERVENTIONS:**				
Foot exercise and education				
Brief advice				
**ASSESSMENTS:**				
Participant characteristics		X		
**Primary outcome measures**				
*Feasibility*				
Number of eligible participants				X
Willingness to enrol				X
Recruitment rate				X
Adherence				X
Logbook completion				X
Adverse effects				X
Dropout rates				X
*Credibility*				
Borkovec and Nau Questionnaire		X	X	
*Acceptability*				
Number of sessions attended				X
Adherence to foot exercises				X
Additional treatment sought				X
Early withdrawal				X
**Secondary outcome measures**				
*Patient reported outcomes*				
First step pain in previous week		X	X	
Worst pain in previous week		X	X	
Average pain in previous week		X	X	
Foot and Ankle Ability Measure		X	X	
Foot Health Status Questionnaire		X	X	
Global Rating of Change Score			X	
Pain Self-Efficacy Scale		X	X	
Active Australia Survey		X	X	
*Physical measurements*				
Intrinsic foot muscle morphology (MRI & ultrasound)		X	X	
Plantar fascia thickness (ultrasound)		X	X	
Intrinsic foot muscle motor performance		X	X	
Foot Posture Index		X	X	
Foot mobility		X	X	
Ankle dorsiflexion range of motion (standing lunge test)		X	X	
Toe flexor strength (dynamometry)		X	X	
Plantar flexor strength (standing heel rise test)		X	X	

^**a**^t_0_ = Baseline, t_1_ = week 12, t_3_ = end of data collection all participants

#### Primary outcome measures

Primary outcomes of this trial will determine the feasibility of conducting a full-scale RCT, as well as the credibility and acceptability of the 12-week foot exercise and education intervention in the treatment of PHP.

1. *Feasibility* will be assessed by: (i) the number of eligible participants (number of eligible participants to number of volunteer responses to trial advertisements); (ii) willingness of volunteers to enrol (number of eligible volunteers to number of enrolled participants); (iii) recruitment rate (number of enrolled participants to the duration of the recruitment period, expressed as participants per week); (iv) adherence with allocated intervention (percentage of physiotherapy visits completed by the participant with respect to the total sessions scheduled; percentage of home training sessions completed by the exercise group with respect to the total training sessions scheduled); (v) log book completion (percentage of training sessions recorded by the participant with respect to the total training sessions to be recorded); (vi) drop-out rate (number of participants who withdrew from the study); and (vii) adverse events.

2. *Credibility* of treatment will be evaluated using the Borkovec and Nau questionnaire [25] which consists of six items related to patient expectations of the allocated treatment (i.e. how much the participant believes that the therapy they are receiving will help to reduce their heel pain). The questionnaire has good test-retest reliability with intra-class correlation coefficients (ICCs) of 0.82 for expectancy and 0.75 for credibility [25]. It will be completed by participants immediately following the first treatment session and at 12-week follow-up.

3. *Acceptability* of the foot exercise intervention and the brief advice intervention will be assessed by: (i) the number of sessions attended as a percentage of the total number of sessions scheduled; (ii) adherence to the foot exercise intervention (percentage of physiotherapy visits completed by the participant with respect to the total sessions scheduled; percentage of home training sessions completed by the participant with respect to the total training sessions scheduled); (iii) additional treatments sought; and (iv) reasons for early withdrawal.

To further explore aspects of feasibility, credibility and acceptability, one-on-one semi-structured interviews will be conducted by the chief investigator. Interviews with participants will be conducted at the conclusion of the 12-week intervention period after completion of follow-up measures and with trial physiotherapists at the conclusion of data collection for all participants. Additional file 8 outlines the question guides.

#### Secondary outcome measures

In the absence of a defined core outcome set for PHP, we will record a comprehensive set of measures that are likely to capture all health related domains of PHP. For example, we will record record a numeric rating scale for first step pain, worst pain and average pain to ensure we are covering the pain aspect of PHP. We will use the Foot and Ankle Ability Measure to capture the disability; the Foot Health Status Questionnaire to evaluate quality of life; the Pain Self-Efficacy Questionnaire to get a sense of the psychological aspects; the Active Australia Survey to gauge how active the participants are; and we will capture foot muscle morphology, foot posture index, foot mobility, toe flexion strength, ankle dorsiflexion, and ankle plantarflexor strength to gain a representation of structural and functional aspects of PHP. These feasibility/practicality of use, response profile (effect size), and completion rates will be used to guide decisions in future trials. For participants with bilateral pain, the foot that the participant reports as having the worst symptoms will be evaluated.

Patient-reported outcome measures will be used to evaluate pain, physical function, physical activity level and pain self-efficacy.

**Fig. 2:**
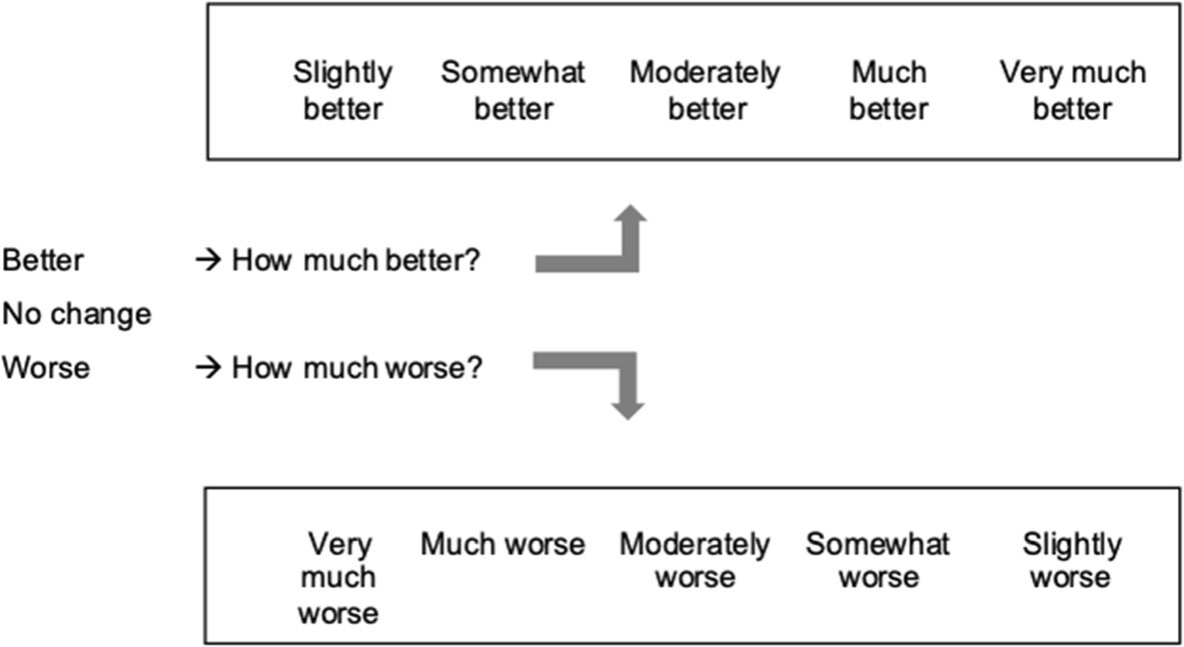
Global Rating of Change Score

1. *Pain severity:* An 11-point numerical rating scale anchored by 0 (no pain at all) and 10 (worst pain imaginable) will be used to rate first step, worst and average level of pain that the participant has experienced in the past week [26]. The numerical rating scale is reported to be more responsive, easier to administer and preferred by patients compared to other pain scales [27]. A 2-point change is reported to be a clinically important difference in individuals with chronic pain [26].

2. *Physical function:* Physical function will be evaluated using the Foot and Ankle Ability Measure and the Foot Health Status Questionnaire. The Foot and Ankle Ability Measure consists of a 21-item activities of daily living subscale and an 8-item sports subscale. Each item is rated by the patient on a 5-point scale as ‘no difficulty’ through to ‘unable to do’. Individual item scores (‘no difficulty’ = 4 through to ‘unable to do’ = 0) are summed and expressed as a percentage of the total score for each subscale (activities of daily living = 84, sports subscale = 32) where 100 represents full function. The Foot and Ankle Ability Measure is a reliable, responsive, and valid measure of physical function for individuals with a broad range of musculoskeletal disorders of the lower leg, foot, and ankle [28]. The minimal clinically important difference is reported as 8 and 9 points for the activities of daily living and sports subscale, respectively [28]. The Foot Health Status Questionnaire consists of 13 items arranged in 3 subscales (foot pain, foot function and general foot health) and converted to a score where 0 represents poor foot health and 100 optimal foot health [29]. The minimal important difference is reported as 14, 7 and 9 points for the foot pain, foot function and general foot health subscales, respectively [30].

3. *Pain self-efficacy:* The Pain Self-Efficacy Questionnaire consists of 10 items, and will be used to assess the confidence participants have in performing activities while in pain [31]. Pain self-efficacy is associated with key outcomes related to chronic pain and to have implications for subsequent functioning [32]. Previous research has reported high test-retest reliability (Pearson’s r = 0.73) [31].

4. *Global rating of change:* Global rating of change score will be obtained using an 11-point scale. Participants will be asked to rate their perceived overall change in condition of their heel pain from the time that they began the study until the present as ‘worse’, ‘no change’ or ‘better’ [33]. If participants indicate ‘worse’, they will then be asked *how much worse* on a 5-point scale from ‘very much worse’ to ‘slightly worse’. Similarly, if participants indicate ‘better’, they will then be asked *how much better* on a 5-point scale of ‘slightly better’ to ‘very much better’ (Fig. 2). The minimal clinical important change is 2-points [33].

5. *Physical activity:* Physical activity level will be evaluated using the Active Australia Survey, which is a short and reliable set of questions designed to measure participation in leisure-time physical activity [34]. The survey has exhibited good reliability with ICCs from 0.71 to 0.86 [34].

Physical measurements will consist of IFM morphology and motor performance, plantar fascia thickness, foot posture and mobility, ankle dorsiflexion range of motion, and toe flexor and plantar-flexor strength.

6. *IFM morphology and composition:* IFM morphology will be measured from high-field MRI and dynamic ultrasound images of the foot, respectively. MRI will permit the evaluation of volume and composition (fat infiltration) of the IFMs. The ability to evaluate whole muscle volumes from MRI will provide quantification of non-uniform changes within a muscle. Although measurements of IFM morphology obtained from ultrasound imaging will be limited to discrete measures of cross-sectional area or thickness, it is included as it is a more accessible, less expensive, clinically feasible measure of IFM morphology than MRI. Inclusion of both imaging modalities will provide insight as to whether the clinically feasible technique of ultrasound imaging has sufficient sensitivity to provide similar information regarding IFM morphology as the gold standard MRI.

A high-resolution fat/water separation imaging sequence (3D; TR 8.68 ms; TE 3.06 and 5.61 ms; 3°; FOV 111 × 223 mm; 0.35 × 0.35 × 0.35 mm; contiguous slices; 434 Hz/Px; TA 11 min 12 s) of the entire foot will be acquired using a whole-body 7 Tesla MRI scanner (Magnetom Terra, Siemens, Erlangen, Germany). Participants will be positioned prone with the ankle in plantarflexion to enable the foot to be positioned within the receiver coil (1Tx 28Rx Knee Coil). For individual IFMs (abductor hallucis, adductor hallucis, flexor digitorum brevis, quadratus plantae, abductor digiti minimi, flexor hallucis brevis, flexor digiti minimi, lumbricals and plantar and dorsal interossei), muscle volume and percentage muscle fat infiltration will be measured from the fat-water images. Regions of interest will be manually contoured for each muscle. Muscle volumes and the percentage of muscle fat infiltration (muscle fat infiltration % = Fat/(Fat+Water)*100) will be calculated from the segmented images for each muscle using established methods [35, 36].

A LOGIQ V2 ultrasound machine (GE Healthcare, Illinois, USA) equipped with a linear array probe (6-12 MHz) will be used to obtain images of the IFMs. Cross-sectional area of the abductor hallucis and thickness of the muscles of the first interstitial space (adductor hallucis, first dorsal interossei and first lumbrical) will be imaged in sitting and standing positions using previously established methods [37]. For abductor hallucis, cross-sectional area will be measured from transverse images obtained on a scanning line drawn in an inferior direction from the most anterior aspect of the medial malleolus. Thickness of the muscles of the first interstitial space will be measured from longitudinal images obtained on a scanning line between the first and second metatarsal bones. Cross-sectional area of flexor digitorum brevis, quadratus plantae and flexor hallucis brevis will be measured from transverse images obtained with the participant in prone with the foot relaxed over the edge of the bed [38]. The thickest portion of the flexor digitorum brevis and quadratus plantae muscles will be located in longitudinal along a scanning line from the medial tubercle of the calcaneus to the third toe, and then the probe rotated to 90 degrees to measure the cross-sectional area. For flexor hallucis brevis the thickest portion of the muscle will be located in longitudinal along the first metatarsal shaft and then the probe rotated to 90 degrees to measure the cross-sectional area. For reproducibility at follow-up, the location of the probe will be recorded as the linear distance from the posterior aspect of the calcaneus. Ultrasound images will be stored and measured offline using medical imaging software (Osirix MD, Pixmeo SARL, Geneva, Switzerland). Test-retest reliability of these measures is high with ICCs 0.81 to 0.98, standard error of measurement (SEM) 0.13 to 0.17cm for thickness and 0.13 to 0.19cm^2^ for cross-sectional area, and minimal detectable change (MDC) of 0.36 to 0.48cm for thickness and 0.14 to 0.53cm^2^ for cross-sectional area [37, 38].

7. *Plantar fascia thickness:* Ultrasound imaging will also be used to assess sagittal thickness of the plantar fascia at the anterior margin of the calcaneus, using previously established methods [39]. Participants will be positioned in prone with the foot relaxed over the edge of the plinth and metatarsophalangeal joints relaxed [40]. Longitudinal images will be obtained 0.5 cm medial to the midline of the plantar aspect of the foot [39]. Test-retest reliability has been established with ICC 0.67 to 0.77 [39]. Based on data provided, SMD is estimated as 0.17 to 0.20 mm, and MDC 0.47 mm to 0.55 mm [39].

8. *IFM motor performance:* Motor performance of four foot movements will be rated by an experienced physiotherapist from video recordings. The foot movements will include (1) great toe extension, (2) lesser toe extension, (3) toe spread, and (4) doming. Each movement will be rated on a scale of 0 to 3 (0 = does not initiate movement or starting position cannot be maintained, 1 = partially completes the exercise, 2 = completes the exercise with compensatory movements, slowness or obvious clumsiness and 3 = completes the exercise with a standard pattern) [41]. Examples of compensatory movements include excessive extrinsic muscle contribution (e.g. able to maintain extension of the interphalangeal joints of the toes), movement out of the intended plane of action or unintended overflow into adjacent planes or joints, such as, pronation or supination of the foot and ankle. Fair to substantial test-retest reliability has been reported (ICCs 0.24 to 0.68) [41]. Following each movement task, perceived difficulty will be assessed by asking the participant to rate the task using a 5-point Likert scale (1 = very easy, 2 = somewhat easy, 3 = neutral, 4 = somewhat difficult and 5 = very difficult) [41]. Slight to substantial test-retest reliability has been reported with ICCs 0.15 to 0.96, SEM 0.4 to 0.7 and MDC of 1.1 to 2.0 [41].

9. *Foot posture:* The Foot Posture Index is an established method of visually classifying static foot posture (normal/pronated/supinated) and is calculated from six criterion- based observations [42]. High test-retest reliability has been previously reported with ICC of 0.98 [43]. SEM is estimated as 0.66 and MDC as 1.83, based on data provided [43].

10. *Foot mobility:* Measures of dorsal arch height and midfoot width will be measured with the participant in standing (weight bearing) and sitting (non-weight bearing), using established methods [44]. The difference between weight bearing and non-weight bearing measurements of midfoot height and midfoot width will be calculated to provide an indication of sagittal plane and frontal plane foot mobility, respectively [44]. In addition, foot mobility magnitude will be calculated from these measures to provide a composite indication of foot mobility. Test-retest reliability is high with ICCs 0.60 to 0.98, SEM 0.06 to 0.13 cm, and MDC 0.16 to 0.35 cm [44].
11. *Ankle dorsiflexion range of motion:* Weight-bearing ankle dorsiflexion range of motion will be measured using a previously established custom device that is based on the standing lunge test [45]. Participants will stand on the device with their second toe, midpoint of the heel and patella of their test leg aligned perpendicular to a movable upright. While maintaining their heel on the floor, participants will be asked to lunge forward at the ankle, moving the upright forward in the sagittal plane. The examiner will monitor the heel during the movement, ensuring that the heel maintains contact with the ground. At the point where the heel just starts to lift, the examiner will stop the movement and record the horizontal distance between the patella and end of the longest toe (millimetres), indicated by the sagittal displacement of the upright. The test will be repeated three times on each limb, with the average of three measures used in analyses. Test-retest reliability is high (ICC 0.99) and the SEM is 1 mm (MDC estimated as 2.8 mm based on data provided) [45].

12. *Toe flexor and plantar flexor strength:* Strength of the great toe flexors and lesser toe flexors will be assessed using a hand held dynamometer (Lafayette Manual Muscle Tester, Lafayette Instrument Co, Indiana, USA). Participants will be seated with their hips and knees in 90 degrees of flexion, ankle joint neutral and metatarsophalangeal joint at the edge of a step so that the toes can be flexed at the metatarsophalangeal joint [46, 47]. For each test the handheld dynamometer will be placed under the proximal phalanx of the great or lesser toes and stabilized/fixed inferiorly (against ground). To bias intrinsic muscle contribution and minimize extrinsic muscle contribution, participants will be instructed to keep their toes straight at the interphalangeal joints. Participant will be instructed to “push the [great or lesser] toe/s into the dynamometer as hard as you can” to achieve a maximum isometric contraction. Each test will be performed three times with the maximum result being selected for data analysis. Data will be normalized to body weight. Following a period of rest, the tests will be repeated with the participant in standing, with the metatarsophalangeal joint at the edge of a step so that the toes can be flexed. Test-retest of toe flexor strength using a hand-held dynamometer is good with ICCs exceeding 0.67 to 0.92, SEM of 18 N and MDC of 49.8 N [41, 48].

Plantar flexor strength will be measured using the Standing Heel Rise Test [49]. Participants will be asked to “lift the heel as high as possible for each heel rise, until no further repetitions can be performed, keeping the knee and trunk straight”. The test will be performed on a level surface, with the participant barefoot, standing on the test limb, with fingertip support on an adjacent wall to assist balance. Speed of the test will be controlled with a metronome at 60 beats per minute. The test will be stopped when the participant: (i) can no longer complete a full range of heel lift; (ii) cannot maintain pace, or knee and trunk alignment or (iii) uses the wall for assistance rather than balance support. The number of heel rises completed will be recorded. Test-retest reliability is excellent (ICC 0.96, SEM 2.2 repetitions, MDC estimated as 6.1 repetitions from data provided) [49].

### Other measures

Participant demographic data (sex, age, occupation and time spent barefoot per day) and body size (height and body mass) will be obtained at baseline for the description of the population.

### Sample size

Due to the exploratory nature of this novel research with the use of MRI and within funding constraints, a sample size of 20 participants (10 per group) will be enrolled into this feasibility study. Sample sizes of this magnitude will best provide estimate parameters for determining a clinical trial that seeks to identify large standardised effect sizes [50].

### Statistical analysis

Data will be analysed on an intention-to-treat basis. Primary outcomes will be presented as descriptive statistics. To explore treatment effects *(secondary aim)*, descriptive data (group means, standard deviation) and mean differences (95% confidence intervals) for within and between-group comparisons will be reported. To provide an indication of the magnitude of observed differences, standardised mean differences (mean difference / pooled standard deviation) will also be calculated. Changes within and between groups will be considered in relation to reported scores of meaningful change and measurement error. The descriptive data and point estimates of treatment effects reported from this study will be used to calculate the required sample size for a full-scale RCT.

## Discussion

PHP is a recurrent and persistent musculoskeletal condition that affects a wide range of individuals. The burden of the condition is significant with low physical activity levels, poor quality of life and higher rates of stress, anxiety and depression [2, 9]. Current evidence to guide selection of treatment options for PHP is limited, with several systematic reviews reporting that treatment effects are small, ineffective when compared to placebo or of short duration only (1–6 weeks) [51–54]. In terms of IFM exercise for PHP, only three superiority trials have been conducted and results provide little evidence to guide the use of exercise for PHP.

Our feasibility study of a prospective assessor-blinded randomised clinical trial will be the first to evaluate the feasibility of a full-scale RCT, as well as treatment response, of a progressive foot focused exercise plus education intervention for the management of PHP, when compared to a wait and see approach (brief advice). The FEET trial will address several limitations of previous investigations, such as exercise programmes with insufficient training volume and intensity, number of exercises, lack of progression and integration to functional positions. The exercise program in this study was developed based on existing biomechanical studies that have reported the function of the IFMs during walking and running [11–13]. Accordingly, the exercises are designed to train the foot for these functional requirements. For example, our program will incorporate varied contraction types (i.e. isometric, concentric and eccentric); functional weight bearing positions; progressive loading via seated, two-leg or single-leg weight bearing or increasing resistance tubing; incorporation of functional tasks (e.g. calf raise to mimic propulsion and squat to mimic stance phase absorption) and rate of loading (e.g. jumping and hopping). The exercise program has been refined through pilot investigations and feedback from individuals with PHP and physiotherapists practicing in the Brisbane region. A novel feature of the FEET trial is the inclusion of a comprehensive set of physical measurements (including IFM morphology), which will help to explore potential mechanisms of effect, whereas existing investigations have focused solely on patient-reported outcomes. An improved understanding of potential mechanisms of effect is a preliminary step towards identifying individuals that may be more suited to this approach.

Although methodological factors have been incorporated into the design of the study to minimise bias (e.g. randomised group allocation via concealed allocation and outcome assessment by a blinded assessor), there are some sources that could not be addressed. For example, it is not possible to blind the participants or the treating physiotherapists to the allocated groups due to the nature of the interventions. We also acknowledge the potential for performance bias due to the structure of the interventions and different contact time with the treating physiotherapist (i.e. one treatment session versus eight treatment sessions). We selected our comparator group (brief advice) to best reflect a wait-and-see approach, given the lack of studies in the literature that compare interventions for PHP to a wait-and-see or minimal intervention. The ‘brief advice’ provided is deliberately intended to provide minimal information for that reason.

The trial will be reported in accordance with the CONSORT statement. The results of this feasibility trial will inform the development of a full-scale RCT including the calculation of required sample size. Future high quality RCTs are required to identify effective interventions for PHP and improve the management of this recurrent and persistent condition.

### Trial status

The study was registered on 11 July 2019. Enrolment of the first participant was 2 August 2019. Data collection commenced on 2 August 2019. The final follow-up measurement was conducted on 31 January 2020.

## Supplementary information

**Additional file 1.** Online screening form

**Additional file 2.** Telephone and physical screening form

**Additional file 3.** Foot Exercise and Education group Logbook

**Additional file 4.** Foot Exercise and Education group Exercise Protocol

**Additional file 5.** Foot Exercise and Education group Handout

**Additional file 6.** Brief Advice group Handout

**Additional file 7.** Brief Advice group Logbook

**Additional file 8.** Question guide for semi-structured interviews

## Data Availability

Data sharing not applicable to this article as no data-sets were generated or analyzed during the current study.

## Abbreviations

CONSORT: Consolidated Standards of Reporting Trials
FEET Trial: Foot Exercise and Education for the Treatment of plantar heel pain
ICC: Intra-class correlation coefficient
IFMs: Intrinsic foot muscles
MDC: Minimal detectable change
MRI: Magnetic resonance imaging
PHP: Plantar heel pain
SEM: Standard error of measurement
SPIRIT: Standard Protocol Items Recommendations For Interventional Trials
