# Supplementary material for: Foot exercise plus education versus wait and see for the treatment of plantar heel pain (FEET trial): a protocol for a feasibility study
Source: J Foot Ankle Res. 2020 May 8;13:20. doi: 10.1186/s13047-020-00384-1 (PMC7206811; doi:10.1186/s13047-020-00384-1)
Supplement: Supplementary file 1 — Additional file 1. Online screening form [file 13047_2020_384_MOESM1_ESM.pdf]

UQ FEET Trial Screening

Thank you for your interest in our research study. The FEET Trial is investigating treatment options for people with heel pain. The following questions are designed to help us determine your eligibility to participate in the trial.

* Required

Contact information

So that we may contact you regarding participation in the study, please provide the following contact information.

1. **What is your name?**

   ____

2. **Please provide the best phone number to contact you on?** *

   ____

3. **Please provide an email address we can contact you on?** *

   ____

4. **Is there a preferred time of day to contact you?**

   ____

5. **Please indicate your sex**

   Mark only one oval.

   - ◯ Female
   - ◯ Male

6. **How old are you?** *

Mark only one oval.

- Under 18 years *Skip to "Survey completed!."*
- Over 18 years

Symptom information

The following questions are related to the foot pain that you have been experiencing.

7. **Is your pain located underneath your foot and at the heel (e.g. somewhere within the blue shading in the picture below)?** *

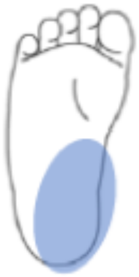

Mark only one oval.

- Yes
- No *Skip to "Survey completed!."*

Untitled Section

8. **How long have you experienced heel pain?** *

Mark only one oval.

- Shorter than 3 months *After the last question in this section, skip to "Survey completed!."*
- Longer than 3 months

9. **Using a number from 0 to 10, where 0 is no pain and 10 is the worst pain imaginable, what number would you use to rate the highest level of your heel pain in the last week?** *

Mark only one oval.

	0	1	2	3	4	5	6	7	8	9	10	
No pain												Worst pain imaginable

10. **Have you had a corticosteroid injection in the past 6 months?**
Mark only one oval.

- ◯ Yes
- ◯ No
- ◯ Not sure

General Health

The following questions relate to your general health, e.g. existing medical conditions

11. **Do you have any of the following? Please tick all that apply.**
Check all that apply.

- ☐ Diabetes
- ☐ Inflammatory systemic disease e.g. rheumatoid arthritis, lupus
- ☐ Cardiac pacemaker
- ☐ Previous surgery to your foot or ankle
- ☐ None
- ☐ Not sure

Survey completed!

Thank you for taking the time to complete these screening questions. Based on your responses and potential eligibility, a researcher will contact you with further information about the study.

Stop filling out this form.

Survey completed!

Based on your responses, you are not eligible to participate in the FEET Trial. Thank your for your time.

Powered by
Google Forms
