# Supplementary material for: Foot exercise plus education versus wait and see for the treatment of plantar heel pain (FEET trial): a protocol for a feasibility study
Source: J Foot Ankle Res. 2020 May 8;13:20. doi: 10.1186/s13047-020-00384-1 (PMC7206811; doi:10.1186/s13047-020-00384-1)
Supplement: Supplementary file 2 — Additional file 2. Telephone and physical screening form [file 13047_2020_384_MOESM2_ESM.pdf]

Participant Screening Form

Title:
Foot **E**xercise and **E**ducation in the **T**reatment of plantar heel pain (FEET Trial): A feasibility trial.

Protocol Number: 2019000772

Principal Investigator:
Dr Melinda Smith, School of Health and Rehabilitation Sciences, The University of Queensland

Associate Investigators:
Prof Bill Vicenzino, School of Health and Rehabilitation Sciences, The University of Queensland
Dr Natalie Collins, School of Health and Rehabilitation Sciences, The University of Queensland
Dr Rebecca Mellor, School of Health and Rehabilitation Sciences, The University of Queensland
Dr Alison Grimaldi, PhysioTec and School of Health and Rehabilitation Sciences, The University of Queensland

Participant Name:	DOB:
Contact email:	Contact phone:

Clinical characteristics of pain:

1) Describe the onset of pain, including the date pain commenced:

2) Describe the *location* of symptoms:

   a) Which is the painful limb:
   ☐ left$_0$ ☐ right$_1$ ☐ both$_2$

*If both feet painful, which is the more painful side? ☐ left$_0$ ☐ right$_1$

   b) Please indicate on the body chart to the right, the location of your pain.

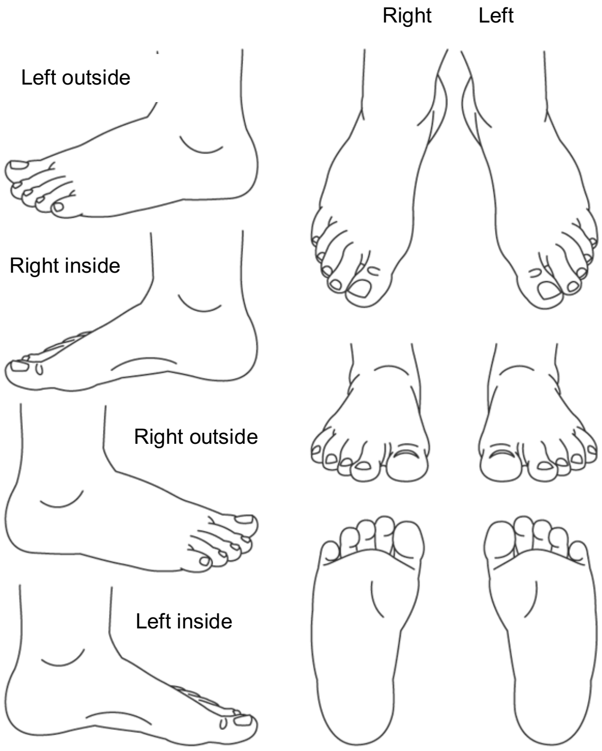

3) Please describe the characteristics of your symptoms:

1. Explain the *nature* of your pain?
   - ☐ sharp$_1$
   - ☐ dull$_2$
   - ☐ ache$_3$
   - ☐ burning$_4$
   - ☐ other$_5$:______________________________

2. Explain the *behaviour* of your pain?
   - ☐ constant$_1$
   - ☐ intermittent$_2$

4) **For the past week,** indicate (circle) the **average** *intensity* of pain:

0 1 2 3 4 5 6 7 8 9 10

No pain ... Worst pain imaginable

5) **For the past week,** indicate (circle) the *intensity* of your **first step pain** (first steps out of bed in the morning or following inactivity):

0 1 2 3 4 5 6 7 8 9 10

No pain ... Worst pain imaginable

6) **For the past week,** indicate (circle) the **worst** *intensity* of pain:

0 1 2 3 4 5 6 7 8 9 10

No pain ... Worst pain imaginable

Indicate the activity related to worst pain: ______________________________

THE UNIVERSITY OF QUEENSLAND
AUSTRALIA

Inclusion Criteria from phone screening (patient interview):

	YES	NO
1. History of heel pain > 3 months	☐	☐
2. Pain worse with initial steps after period of inactivity	☐	☐
3. First step heel pain of > 3/10 on NRS	☐	☐

Exclusion Criteria from phone screening (patient interview):

	YES	NO	Please specify details:
1. Below 18 years of age	☐	☐	
2. Corticosteroid injection for plantar fasciopathy within the previous six months	☐	☐	
2. Cardiac condition	☐	☐	
3. Neurological condition	☐	☐	
4. Diabetes	☐	☐	
5. Auto-immune disorder	☐	☐	
6. Foot pathology/deformities (e.g. nerve impingement, stress fracture, hallux valgus)	☐	☐	
7. Previous foot surgery	☐	☐	
8. Other lower limb pain/injury (preceding six months)	☐	☐	
9. Unsafe or unwilling to undergo MRI (please see detailed MRI screening on page below)	☐	☐	

Suitable for study based on phone screening (patient interview):

☐ **Yes** ☐ **No** **Date:**

Physical examination scheduled for (date/time):

THE UNIVERSITY OF QUEENSLAND
AUSTRALIA

***MRI screening questions**: Do you have (or have ever had):*		
Cardiac pacemaker or artificial heart valve	☐ No	☐ Yes
Syringe driver (i.e. insulin infusion pump)	☐ No	☐ Yes
Brain aneurysm clip or aortic clip	☐ No	☐ Yes
Stent, coil or catheter	☐ No	☐ Yes
Electrical neurostimulators (DBS)	☐ No	☐ Yes
Metal mesh implants / clips / wire sutures	☐ No	☐ Yes
Medicated skin patches	☐ No	☐ Yes
Hearing aid / implant	☐ No	☐ Yes
Glass eye	☐ No	☐ Yes
Joint replacement	☐ No	☐ Yes
Bullet / shrapnel wound	☐ No	☐ Yes
Metal fragments in eye, head, skin	☐ No	☐ Yes
Artificial limb	☐ No	☐ Yes
Do you work with metals?	☐ No	☐ Yes
Do you suffer claustrophobia?	☐ No	☐ Yes
Could you be pregnant?	☐ No	☐ Yes
Do you have an IUD?	☐ No	☐ Yes
Fractured bones treated with metal?	☐ No	☐ Yes
Have you had any surgery?	☐ No	☐ Yes
A shunt – spinal or ventricular?	☐ No	☐ Yes
Do you have any tattoos?	☐ No	☐ Yes
Dental bridge, dentures or retainer?	☐ No	☐ Yes
Do you have a history of kidney disease / disorder?	☐ No	☐ Yes
Do you have asthma?	☐ No	☐ Yes
If you answered 'yes' to any of the above, please provide details:		

Cleared for MRI: ☐ No ☐ Yes

Inclusion Criteria from physical examination:

	YES	NO
1. Pain on palpation of the medial calcaneal tubercle or proximal plantar fascia	☐	☐
2. Plantar fascia thickness >4mm from ultrasound image measurement	☐	☐

Suitable for study following patient interview and physical examination:

☐ **Yes** ☐ **No** **Date:**

Baseline testing scheduled for (date/time):
