# Supplementary material for: Foot exercise plus education versus wait and see for the treatment of plantar heel pain (FEET trial): a protocol for a feasibility study
Source: J Foot Ankle Res. 2020 May 8;13:20. doi: 10.1186/s13047-020-00384-1 (PMC7206811; doi:10.1186/s13047-020-00384-1)
Supplement: Supplementary file 3 — Additional file 3. Foot Exercise and Education group Logbook [file 13047_2020_384_MOESM3_ESM.pdf]

Participant Logbook – *Foot exercise and education*

Title: **F**oot **E**xercise and **E**ducation in the **T**reatment of plantar heel pain (FEET Trial): A feasibility trial.
Protocol Number: **2019000772**
Principal Investigator: Dr Melinda Smith, School of Health and Rehabilitation Sciences, The University of Queensland
Associate Investigators: Prof Bill Vicenzino, School of Health and Rehabilitation Sciences, The University of Queensland
Dr Natalie Collins, School of Health and Rehabilitation Sciences, The University of Queensland
Dr Rebecca Mellor, School of Health and Rehabilitation Sciences, The University of Queensland
Dr Alison Grimaldi, PhysioTec and School of Health and Rehabilitation Sciences, The University of Queensland

Please use this logbook to record the following information daily for the 12 weeks of the intervention period.

Week 1 Date of Week Beginning: ______________________________ **Participant ID:** ___________________

Exercise	Monday	Tuesday	Wednesday	Thursday	Friday	Saturday	Sunday
**DAILY EXERCISES**							
Doming + long toe push 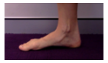	Reps:	Reps:	Reps:	Reps:	Reps:	Reps:	Reps:
Toe spread 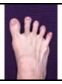	Reps:	Reps:	Reps:	Reps:	Reps:	Reps:	Reps:
Great toe extension 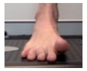	Reps:	Reps:	Reps:	Reps:	Reps:	Reps:	Reps:
Less toe extension 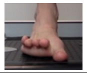	Reps:	Reps:	Reps:	Reps:	Reps:	Reps:	Reps:
**Other relevant information**							
Details of other activities performed							
Details of any adverse events							
Details of treatment outside of study							

For this past week, indicate (circle) the *intensity* of your **<u>first step pain</u>** (first steps out of bed in the morning): (Date completed: ___________________)

0 1 2 3 4 5 6 7 8 9 10

No pain — Worst pain imaginable

Week 2 Date of Week Beginning: ______________________ **Participant ID:** ______________

Exercise	Monday	Tuesday	Wednesday	Thursday	Friday	Saturday	Sunday
**DAILY EXERCISES**							
Doming + long toe push 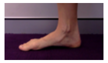	Reps:	Reps:	Reps:	Reps:	Reps:	Reps:	Reps:
Toe spread 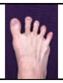	Reps:	Reps:	Reps:	Reps:	Reps:	Reps:	Reps:
Great toe extension 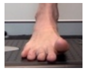	Reps:	Reps:	Reps:	Reps:	Reps:	Reps:	Reps:
Less toe extension 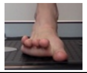	Reps:	Reps:	Reps:	Reps:	Reps:	Reps:	Reps:
Sit to stand 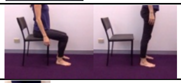	Reps:	Reps:	Reps:	Reps:	Reps:	Reps:	Reps:
Single leg balance 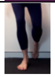	Reps:	Reps:	Reps:	Reps:	Reps:	Reps:	Reps:
**EXERCISES TO PERFORM 3 TIMES A WEEK**							
Doming 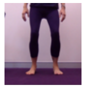 2 leg squat 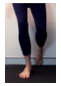 1 leg squat 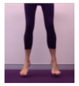 Jump or hop	Reps:<br>Sets:	Reps:<br>Sets:	Reps:<br>Sets:	Reps:<br>Sets:	Reps:<br>Sets:	Reps:<br>Sets:	Reps:<br>Sets:
Long toe push 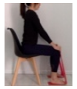 Sit 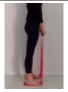 Stand 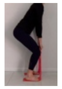 Squat	Reps:<br>Sets:	Reps:<br>Sets:	Reps:<br>Sets:	Reps:<br>Sets:	Reps:<br>Sets:	Reps:<br>Sets:	Reps:<br>Sets:
Seated heel raise 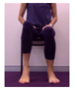 Sit 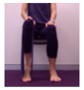 Band max reps 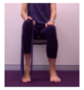 Band max reps	Reps:<br>Sets:	Reps:<br>Sets:	Reps:<br>Sets:	Reps:<br>Sets:	Reps:<br>Sets:	Reps:<br>Sets:	Reps:<br>Sets:
Standing heel raise 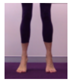 2 leg 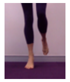 1 leg 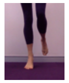 1 leg max reps	Reps:<br>Sets:	Reps:<br>Sets:	Reps:<br>Sets:	Reps:<br>Sets:	Reps:<br>Sets:	Reps:<br>Sets:	Reps:<br>Sets:
**Other relevant information**							
Details of other activities performed							
Details of any adverse events							
Details of treatment outside of study							

For this past week, indicate (circle) the *intensity* of your **<u>first step pain</u>** (first steps out of bed in the morning): (Date completed: ______________)

0 1 2 3 4 5 6 7 8 9 10

No pain — Worst pain imaginable

Week 3 Date of Week Beginning: ______________________ **Participant ID:** ______________

Exercise				Monday	Tuesday	Wednesday	Thursday	Friday	Saturday	Sunday
**DAILY EXERCISES**										
Doming + long toe push	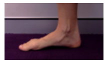			Reps:	Reps:	Reps:	Reps:	Reps:	Reps:	Reps:
Toe spread	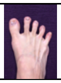			Reps:	Reps:	Reps:	Reps:	Reps:	Reps:	Reps:
Great toe extension	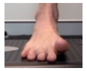			Reps:	Reps:	Reps:	Reps:	Reps:	Reps:	Reps:
Less toe extension	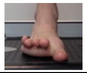			Reps:	Reps:	Reps:	Reps:	Reps:	Reps:	Reps:
Sit to stand	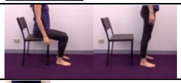			Reps:	Reps:	Reps:	Reps:	Reps:	Reps:	Reps:
Single leg balance	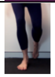			Reps:	Reps:	Reps:	Reps:	Reps:	Reps:	Reps:
**EXERCISES TO PERFORM 3 TIMES A WEEK**										
Doming	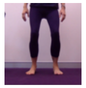 2 leg squat	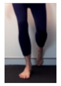 1 leg squat	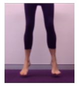 Jump or hop	Reps:<br>Sets:	Reps:<br>Sets:	Reps:<br>Sets:	Reps:<br>Sets:	Reps:<br>Sets:	Reps:<br>Sets:	Reps:<br>Sets:
Long toe push	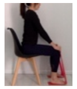 Sit	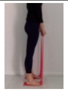 Stand	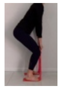 Squat	Reps:<br>Sets:	Reps:<br>Sets:	Reps:<br>Sets:	Reps:<br>Sets:	Reps:<br>Sets:	Reps:<br>Sets:	Reps:<br>Sets:
Seated heel raise	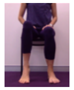 Sit	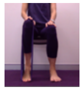 Band max reps	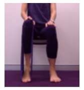 Band max reps	Reps:<br>Sets:	Reps:<br>Sets:	Reps:<br>Sets:	Reps:<br>Sets:	Reps:<br>Sets:	Reps:<br>Sets:	Reps:<br>Sets:
Standing heel raise	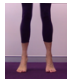 2 leg	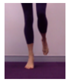 1 leg	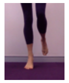 1 leg max reps	Reps:<br>Sets:	Reps:<br>Sets:	Reps:<br>Sets:	Reps:<br>Sets:	Reps:<br>Sets:	Reps:<br>Sets:	Reps:<br>Sets:
**Other relevant information**										
Details of other activities performed										
Details of any adverse events										
Details of treatment outside of study										

For this past week, indicate (circle) the *intensity* of your **first step pain** (first steps out of bed in the morning): (Date completed: ______________)

0 1 2 3 4 5 6 7 8 9 10

No pain — Worst pain imaginable

Week 4 Date of Week Beginning: ______________________________ **Participant ID:** ___________________

Exercise				Monday	Tuesday	Wednesday	Thursday	Friday	Saturday	Sunday
**DAILY EXERCISES**										
Doming + long toe push	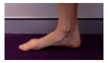			Reps:	Reps:	Reps:	Reps:	Reps:	Reps:	Reps:
Toe spread	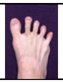			Reps:	Reps:	Reps:	Reps:	Reps:	Reps:	Reps:
Great toe extension	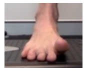			Reps:	Reps:	Reps:	Reps:	Reps:	Reps:	Reps:
Less toe extension	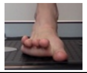			Reps:	Reps:	Reps:	Reps:	Reps:	Reps:	Reps:
Sit to stand	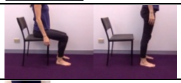			Reps:	Reps:	Reps:	Reps:	Reps:	Reps:	Reps:
Single leg balance	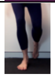			Reps:	Reps:	Reps:	Reps:	Reps:	Reps:	Reps:
**EXERCISES TO PERFORM 3 TIMES A WEEK**										
Doming	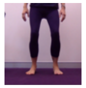 2 leg squat	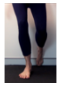 1 leg squat	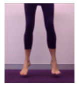 Jump or hop	Reps:<br>Sets:	Reps:<br>Sets:	Reps:<br>Sets:	Reps:<br>Sets:	Reps:<br>Sets:	Reps:<br>Sets:	Reps:<br>Sets:
Long toe push	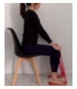 Sit	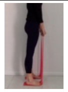 Stand	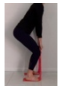 Squat	Reps:<br>Sets:	Reps:<br>Sets:	Reps:<br>Sets:	Reps:<br>Sets:	Reps:<br>Sets:	Reps:<br>Sets:	Reps:<br>Sets:
Seated heel raise	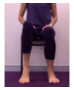 Sit	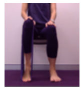 Band max reps	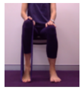 Band max reps	Reps:<br>Sets:	Reps:<br>Sets:	Reps:<br>Sets:	Reps:<br>Sets:	Reps:<br>Sets:	Reps:<br>Sets:	Reps:<br>Sets:
Standing heel raise	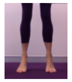 2 leg	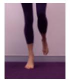 1 leg	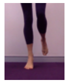 1 leg max reps	Reps:<br>Sets:	Reps:<br>Sets:	Reps:<br>Sets:	Reps:<br>Sets:	Reps:<br>Sets:	Reps:<br>Sets:	Reps:<br>Sets:
**Other relevant information**										
Details of other activities performed										
Details of any adverse events										
Details of treatment outside of study										

For this past week, indicate (circle) the *intensity* of your **first step pain** (first steps out of bed in the morning): (Date completed: ___________________)

0 1 2 3 4 5 6 7 8 9 10

No pain … Worst pain imaginable

Week 5 Date of Week Beginning: ______________________________ **Participant ID:** ___________________

Exercise	Monday	Tuesday	Wednesday	Thursday	Friday	Saturday	Sunday
**DAILY EXERCISES**							
Doming + long toe push 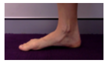	Reps:	Reps:	Reps:	Reps:	Reps:	Reps:	Reps:
Toe spread 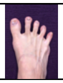	Reps:	Reps:	Reps:	Reps:	Reps:	Reps:	Reps:
Great toe extension 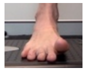	Reps:	Reps:	Reps:	Reps:	Reps:	Reps:	Reps:
Less toe extension 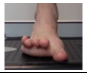	Reps:	Reps:	Reps:	Reps:	Reps:	Reps:	Reps:
Sit to stand 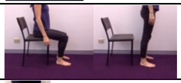	Reps:	Reps:	Reps:	Reps:	Reps:	Reps:	Reps:
Single leg balance 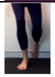	Reps:	Reps:	Reps:	Reps:	Reps:	Reps:	Reps:
**EXERCISES TO PERFORM 3 TIMES A WEEK**							
Doming | 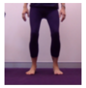 2 leg squat | 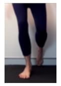 1 leg squat | 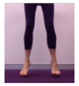 Jump or hop	Reps:<br>Sets:	Reps:<br>Sets:	Reps:<br>Sets:	Reps:<br>Sets:	Reps:<br>Sets:	Reps:<br>Sets:	Reps:<br>Sets:
Long toe push | 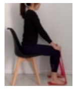 Sit | 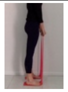 Stand | 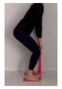 Squat	Reps:<br>Sets:	Reps:<br>Sets:	Reps:<br>Sets:	Reps:<br>Sets:	Reps:<br>Sets:	Reps:<br>Sets:	Reps:<br>Sets:
Seated heel raise | 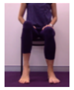 Sit | 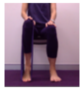 Band max reps | 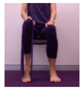 Band max reps	Reps:<br>Sets:	Reps:<br>Sets:	Reps:<br>Sets:	Reps:<br>Sets:	Reps:<br>Sets:	Reps:<br>Sets:	Reps:<br>Sets:
Standing heel raise | 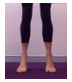 2 leg | 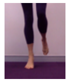 1 leg | 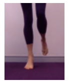 1 leg max reps	Reps:<br>Sets:	Reps:<br>Sets:	Reps:<br>Sets:	Reps:<br>Sets:	Reps:<br>Sets:	Reps:<br>Sets:	Reps:<br>Sets:
**Other relevant information**							
Details of other activities performed							
Details of any adverse events							
Details of treatment outside of study							

For this past week, indicate (circle) the *intensity* of your **first step pain** (first steps out of bed in the morning): (Date completed: ____________________)

0 1 2 3 4 5 6 7 8 9 10

No pain ... Worst pain imaginable

Week 6 Date of Week Beginning: ______________________________ **Participant ID:** ___________________

Exercise				Monday	Tuesday	Wednesday	Thursday	Friday	Saturday	Sunday
**DAILY EXERCISES**										
Doming + long toe push 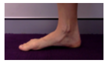				Reps:	Reps:	Reps:	Reps:	Reps:	Reps:	Reps:
Toe spread 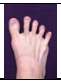				Reps:	Reps:	Reps:	Reps:	Reps:	Reps:	Reps:
Great toe extension 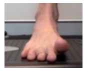				Reps:	Reps:	Reps:	Reps:	Reps:	Reps:	Reps:
Less toe extension 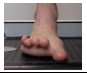				Reps:	Reps:	Reps:	Reps:	Reps:	Reps:	Reps:
Sit to stand 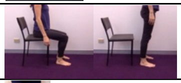				Reps:	Reps:	Reps:	Reps:	Reps:	Reps:	Reps:
Single leg balance 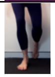				Reps:	Reps:	Reps:	Reps:	Reps:	Reps:	Reps:
**EXERCISES TO PERFORM 3 TIMES A WEEK**										
Doming	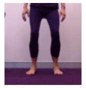 2 leg squat	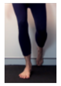 1 leg squat	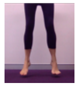 Jump or hop	Reps:<br>Sets:	Reps:<br>Sets:	Reps:<br>Sets:	Reps:<br>Sets:	Reps:<br>Sets:	Reps:<br>Sets:	Reps:<br>Sets:
Long toe push	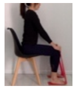 Sit	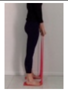 Stand	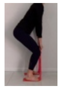 Squat	Reps:<br>Sets:	Reps:<br>Sets:	Reps:<br>Sets:	Reps:<br>Sets:	Reps:<br>Sets:	Reps:<br>Sets:	Reps:<br>Sets:
Seated heel raise	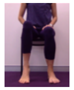 Sit	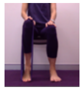 Band max reps	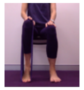 Band max reps	Reps:<br>Sets:	Reps:<br>Sets:	Reps:<br>Sets:	Reps:<br>Sets:	Reps:<br>Sets:	Reps:<br>Sets:	Reps:<br>Sets:
Standing heel raise	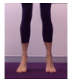 2 leg	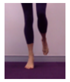 1 leg	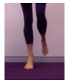 1 leg max reps	Reps:<br>Sets:	Reps:<br>Sets:	Reps:<br>Sets:	Reps:<br>Sets:	Reps:<br>Sets:	Reps:<br>Sets:	Reps:<br>Sets:
**Other relevant information**										
Details of other activities performed										
Details of any adverse events										
Details of treatment outside of study										

For this past week, indicate (circle) the *intensity* of your **first step pain** (first steps out of bed in the morning): (Date completed: ____________________)

0 1 2 3 4 5 6 7 8 9 10

No pain ... Worst pain imaginable

Week 7 Date of Week Beginning: ________________________________ **Participant ID:** ____________________

Exercise				Monday	Tuesday	Wednesday	Thursday	Friday	Saturday	Sunday
**DAILY EXERCISES**										
Doming + long toe push	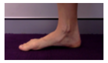			Reps:	Reps:	Reps:	Reps:	Reps:	Reps:	Reps:
Toe spread	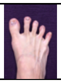			Reps:	Reps:	Reps:	Reps:	Reps:	Reps:	Reps:
Great toe extension	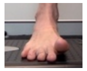			Reps:	Reps:	Reps:	Reps:	Reps:	Reps:	Reps:
Less toe extension	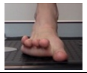			Reps:	Reps:	Reps:	Reps:	Reps:	Reps:	Reps:
Sit to stand	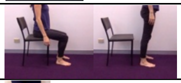			Reps:	Reps:	Reps:	Reps:	Reps:	Reps:	Reps:
Single leg balance	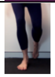			Reps:	Reps:	Reps:	Reps:	Reps:	Reps:	Reps:
**EXERCISES TO PERFORM 3 TIMES A WEEK**										
Doming	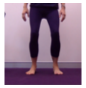 2 leg squat	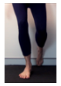 1 leg squat	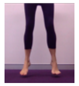 Jump or hop	Reps:<br>Sets:	Reps:<br>Sets:	Reps:<br>Sets:	Reps:<br>Sets:	Reps:<br>Sets:	Reps:<br>Sets:	Reps:<br>Sets:
Long toe push	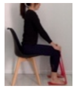 Sit	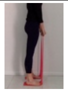 Stand	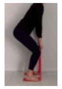 Squat	Reps:<br>Sets:	Reps:<br>Sets:	Reps:<br>Sets:	Reps:<br>Sets:	Reps:<br>Sets:	Reps:<br>Sets:	Reps:<br>Sets:
Seated heel raise	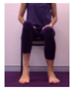 Sit	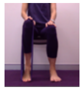 Band max reps	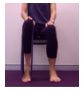 Band max reps	Reps:<br>Sets:	Reps:<br>Sets:	Reps:<br>Sets:	Reps:<br>Sets:	Reps:<br>Sets:	Reps:<br>Sets:	Reps:<br>Sets:
Standing heel raise	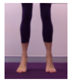 2 leg	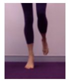 1 leg	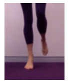 1 leg max reps	Reps:<br>Sets:	Reps:<br>Sets:	Reps:<br>Sets:	Reps:<br>Sets:	Reps:<br>Sets:	Reps:<br>Sets:	Reps:<br>Sets:
**Other relevant information**										
Details of other activities performed										
Details of any adverse events										
Details of treatment outside of study										

For this past week, indicate (circle) the *intensity* of your **<u>first step pain</u>** (first steps out of bed in the morning): (Date completed: ____________________)

0 1 2 3 4 5 6 7 8 9 10

No pain — Worst pain imaginable

Week 8 Date of Week Beginning: ______________________________ **Participant ID:** ___________________

Exercise				Monday	Tuesday	Wednesday	Thursday	Friday	Saturday	Sunday
**DAILY EXERCISES**										
Doming + long toe push	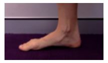			Reps:	Reps:	Reps:	Reps:	Reps:	Reps:	Reps:
Toe spread	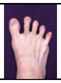			Reps:	Reps:	Reps:	Reps:	Reps:	Reps:	Reps:
Great toe extension	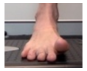			Reps:	Reps:	Reps:	Reps:	Reps:	Reps:	Reps:
Less toe extension	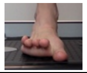			Reps:	Reps:	Reps:	Reps:	Reps:	Reps:	Reps:
Sit to stand	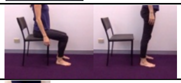			Reps:	Reps:	Reps:	Reps:	Reps:	Reps:	Reps:
Single leg balance	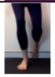			Reps:	Reps:	Reps:	Reps:	Reps:	Reps:	Reps:
**EXERCISES TO PERFORM 3 TIMES A WEEK**										
Doming	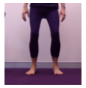 2 leg squat	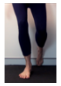 1 leg squat	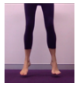 Jump or hop	Reps: Sets:	Reps: Sets:	Reps: Sets:	Reps: Sets:	Reps: Sets:	Reps: Sets:	Reps: Sets:
Long toe push	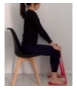 Sit	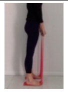 Stand	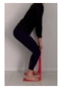 Squat	Reps: Sets:	Reps: Sets:	Reps: Sets:	Reps: Sets:	Reps: Sets:	Reps: Sets:	Reps: Sets:
Seated heel raise	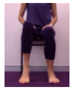 Sit	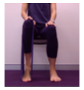 Band max reps	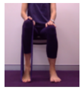 Band max reps	Reps: Sets:	Reps: Sets:	Reps: Sets:	Reps: Sets:	Reps: Sets:	Reps: Sets:	Reps: Sets:
Standing heel raise	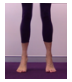 2 leg	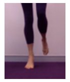 1 leg	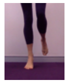 1 leg max reps	Reps: Sets:	Reps: Sets:	Reps: Sets:	Reps: Sets:	Reps: Sets:	Reps: Sets:	Reps: Sets:
**Other relevant information**										
Details of other activities performed										
Details of any adverse events										
Details of treatment outside of study										

For this past week, indicate (circle) the *intensity* of your **first step pain** (first steps out of bed in the morning): (Date completed: ___________________)

0 1 2 3 4 5 6 7 8 9 10

No pain — Worst pain imaginable

Week 9 Date of Week Beginning: ______________________ **Participant ID:** ______________

Exercise	Monday	Tuesday	Wednesday	Thursday	Friday	Saturday	Sunday
**DAILY EXERCISES**							
Doming + long toe push 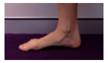	Reps:	Reps:	Reps:	Reps:	Reps:	Reps:	Reps:
Toe spread 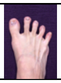	Reps:	Reps:	Reps:	Reps:	Reps:	Reps:	Reps:
Great toe extension 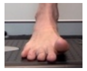	Reps:	Reps:	Reps:	Reps:	Reps:	Reps:	Reps:
Less toe extension 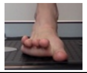	Reps:	Reps:	Reps:	Reps:	Reps:	Reps:	Reps:
Sit to stand 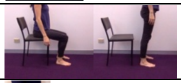	Reps:	Reps:	Reps:	Reps:	Reps:	Reps:	Reps:
Single leg balance 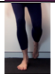	Reps:	Reps:	Reps:	Reps:	Reps:	Reps:	Reps:
**EXERCISES TO PERFORM 3 TIMES A WEEK**							
Doming 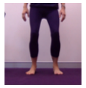 2 leg squat 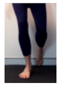 1 leg squat 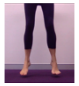 Jump or hop	Reps:<br>Sets:	Reps:<br>Sets:	Reps:<br>Sets:	Reps:<br>Sets:	Reps:<br>Sets:	Reps:<br>Sets:	Reps:<br>Sets:
Long toe push 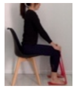 Sit 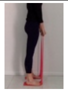 Stand 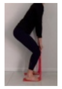 Squat	Reps:<br>Sets:	Reps:<br>Sets:	Reps:<br>Sets:	Reps:<br>Sets:	Reps:<br>Sets:	Reps:<br>Sets:	Reps:<br>Sets:
Seated heel raise 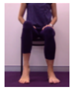 Sit 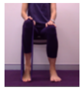 Band max reps 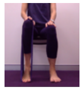 Band max reps	Reps:<br>Sets:	Reps:<br>Sets:	Reps:<br>Sets:	Reps:<br>Sets:	Reps:<br>Sets:	Reps:<br>Sets:	Reps:<br>Sets:
Standing heel raise 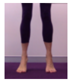 2 leg 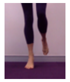 1 leg 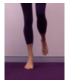 1 leg max reps	Reps:<br>Sets:	Reps:<br>Sets:	Reps:<br>Sets:	Reps:<br>Sets:	Reps:<br>Sets:	Reps:<br>Sets:	Reps:<br>Sets:
**Other relevant information**							
Details of other activities performed							
Details of any adverse events							
Details of treatment outside of study							

For this past week, indicate (circle) the *intensity* of your **first step pain** (first steps out of bed in the morning): (Date completed: ______________)

0 1 2 3 4 5 6 7 8 9 10

No pain — Worst pain imaginable

Week 10 Date of Week Beginning: ______________________ **Participant ID:** ______________

Exercise				Monday	Tuesday	Wednesday	Thursday	Friday	Saturday	Sunday
**DAILY EXERCISES**										
Doming + long toe push	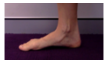			Reps:	Reps:	Reps:	Reps:	Reps:	Reps:	Reps:
Toe spread	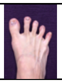			Reps:	Reps:	Reps:	Reps:	Reps:	Reps:	Reps:
Great toe extension	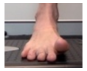			Reps:	Reps:	Reps:	Reps:	Reps:	Reps:	Reps:
Less toe extension	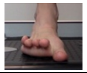			Reps:	Reps:	Reps:	Reps:	Reps:	Reps:	Reps:
Sit to stand	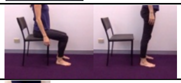			Reps:	Reps:	Reps:	Reps:	Reps:	Reps:	Reps:
Single leg balance	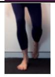			Reps:	Reps:	Reps:	Reps:	Reps:	Reps:	Reps:
**EXERCISES TO PERFORM 3 TIMES A WEEK**										
Doming	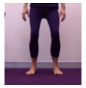 2 leg squat	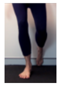 1 leg squat	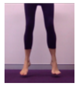 Jump or hop	Reps:<br>Sets:	Reps:<br>Sets:	Reps:<br>Sets:	Reps:<br>Sets:	Reps:<br>Sets:	Reps:<br>Sets:	Reps:<br>Sets:
Long toe push	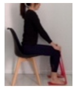 Sit	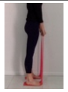 Stand	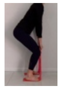 Squat	Reps:<br>Sets:	Reps:<br>Sets:	Reps:<br>Sets:	Reps:<br>Sets:	Reps:<br>Sets:	Reps:<br>Sets:	Reps:<br>Sets:
Seated heel raise	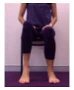 Sit	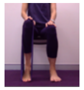 Band max reps	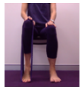 Band max reps	Reps:<br>Sets:	Reps:<br>Sets:	Reps:<br>Sets:	Reps:<br>Sets:	Reps:<br>Sets:	Reps:<br>Sets:	Reps:<br>Sets:
Standing heel raise	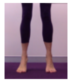 2 leg	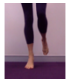 1 leg	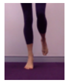 1 leg max reps	Reps:<br>Sets:	Reps:<br>Sets:	Reps:<br>Sets:	Reps:<br>Sets:	Reps:<br>Sets:	Reps:<br>Sets:	Reps:<br>Sets:
**Other relevant information**										
Details of other activities performed										
Details of any adverse events										
Details of treatment outside of study										

For this past week, indicate (circle) the *intensity* of your **first step pain** (first steps out of bed in the morning): (Date completed: ________________)

0 1 2 3 4 5 6 7 8 9 10

No pain ... Worst pain imaginable

Week 11 Date of Week Beginning: ______________________________ **Participant ID:** ___________________

Exercise				Monday	Tuesday	Wednesday	Thursday	Friday	Saturday	Sunday
**DAILY EXERCISES**										
Doming + long toe push	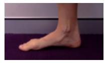			Reps:	Reps:	Reps:	Reps:	Reps:	Reps:	Reps:
Toe spread	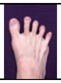			Reps:	Reps:	Reps:	Reps:	Reps:	Reps:	Reps:
Great toe extension	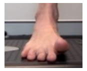			Reps:	Reps:	Reps:	Reps:	Reps:	Reps:	Reps:
Less toe extension	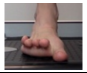			Reps:	Reps:	Reps:	Reps:	Reps:	Reps:	Reps:
Sit to stand	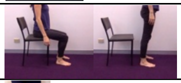			Reps:	Reps:	Reps:	Reps:	Reps:	Reps:	Reps:
Single leg balance	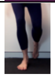			Reps:	Reps:	Reps:	Reps:	Reps:	Reps:	Reps:
**EXERCISES TO PERFORM 3 TIMES A WEEK**										
Doming	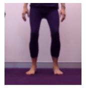 2 leg squat	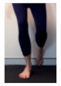 1 leg squat	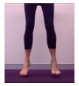 Jump or hop	Reps:<br>Sets:	Reps:<br>Sets:	Reps:<br>Sets:	Reps:<br>Sets:	Reps:<br>Sets:	Reps:<br>Sets:	Reps:<br>Sets:
Long toe push	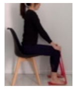 Sit	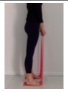 Stand	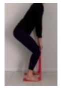 Squat	Reps:<br>Sets:	Reps:<br>Sets:	Reps:<br>Sets:	Reps:<br>Sets:	Reps:<br>Sets:	Reps:<br>Sets:	Reps:<br>Sets:
Seated heel raise	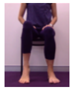 Sit	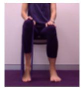 Band max reps	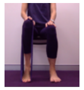 Band max reps	Reps:<br>Sets:	Reps:<br>Sets:	Reps:<br>Sets:	Reps:<br>Sets:	Reps:<br>Sets:	Reps:<br>Sets:	Reps:<br>Sets:
Standing heel raise	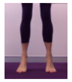 2 leg	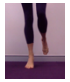 1 leg	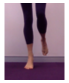 1 leg max reps	Reps:<br>Sets:	Reps:<br>Sets:	Reps:<br>Sets:	Reps:<br>Sets:	Reps:<br>Sets:	Reps:<br>Sets:	Reps:<br>Sets:
**Other relevant information**										
Details of other activities performed										
Details of any adverse events										
Details of treatment outside of study										

For this past week, indicate (circle) the *intensity* of your **first step pain** (first steps out of bed in the morning): (Date completed: ___________________)

0 1 2 3 4 5 6 7 8 9 10

No pain ... Worst pain imaginable

Week 12 Date of Week Beginning: ______________________ **Participant ID:** ______________

Exercise				Monday	Tuesday	Wednesday	Thursday	Friday	Saturday	Sunday
**DAILY EXERCISES**										
Doming + long toe push	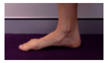			Reps:	Reps:	Reps:	Reps:	Reps:	Reps:	Reps:
Toe spread	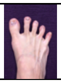			Reps:	Reps:	Reps:	Reps:	Reps:	Reps:	Reps:
Great toe extension	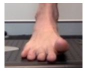			Reps:	Reps:	Reps:	Reps:	Reps:	Reps:	Reps:
Less toe extension	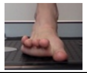			Reps:	Reps:	Reps:	Reps:	Reps:	Reps:	Reps:
Sit to stand	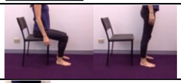			Reps:	Reps:	Reps:	Reps:	Reps:	Reps:	Reps:
Single leg balance	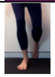			Reps:	Reps:	Reps:	Reps:	Reps:	Reps:	Reps:
**EXERCISES TO PERFORM 3 TIMES A WEEK**										
Doming	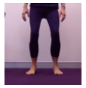 2 leg squat	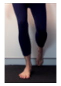 1 leg squat	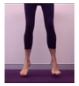 Jump or hop	Reps:<br>Sets:	Reps:<br>Sets:	Reps:<br>Sets:	Reps:<br>Sets:	Reps:<br>Sets:	Reps:<br>Sets:	Reps:<br>Sets:
Long toe push	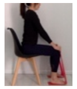 Sit	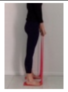 Stand	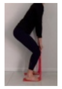 Squat	Reps:<br>Sets:	Reps:<br>Sets:	Reps:<br>Sets:	Reps:<br>Sets:	Reps:<br>Sets:	Reps:<br>Sets:	Reps:<br>Sets:
Seated heel raise	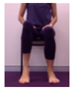 Sit	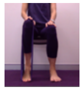 Band max reps	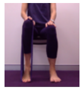 Band max reps	Reps:<br>Sets:	Reps:<br>Sets:	Reps:<br>Sets:	Reps:<br>Sets:	Reps:<br>Sets:	Reps:<br>Sets:	Reps:<br>Sets:
Standing heel raise	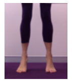 2 leg	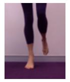 1 leg	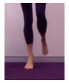 1 leg max reps	Reps:<br>Sets:	Reps:<br>Sets:	Reps:<br>Sets:	Reps:<br>Sets:	Reps:<br>Sets:	Reps:<br>Sets:	Reps:<br>Sets:
**Other relevant information**										
Details of other activities performed										
Details of any adverse events										
Details of treatment outside of study										

For this past week, indicate (circle) the *intensity* of your **first step pain** (first steps out of bed in the morning): (Date completed: ______________)

0 1 2 3 4 5 6 7 8 9 10

No pain ... Worst pain imaginable
