# Supplementary material for: Foot exercise plus education versus wait and see for the treatment of plantar heel pain (FEET trial): a protocol for a feasibility study
Source: J Foot Ankle Res. 2020 May 8;13:20. doi: 10.1186/s13047-020-00384-1 (PMC7206811; doi:10.1186/s13047-020-00384-1)
Supplement: Supplementary file 4 — Additional file 4. Foot Exercise and Education group Exercise Protocol [file 13047_2020_384_MOESM4_ESM.pdf]

Protocol for targeted foot muscle training

Title:
Foot **E**xercise and **E**ducation in the **T**reatment of plantar heel pain (FEET Trial): A feasibility trial.

Protocol Number: 2019000772

Principal Investigator:
Dr Melinda Smith, School of Health and Rehabilitation Sciences, The University of Queensland

Associate Investigators:
Prof Bill Vicenzino, School of Health and Rehabilitation Sciences, The University of Queensland
Dr Natalie Collins, School of Health and Rehabilitation Sciences, The University of Queensland
Dr Rebecca Mellor, School of Health and Rehabilitation Sciences, The University of Queensland
Dr Alison Grimaldi, PhysioTec and School of Health and Rehabilitation Sciences, The University of Queensland

---

The 12 week foot muscle training programme comprises 2 components:

1) **Daily exercises**: Will be performed daily throughout the 12 week intervention and consists of 4 movements that are isolated to the foot intrinsics and 2 movements that incorporate the whole lower limb.

2) **Exercises to perform 3 times a week**: 4 exercises will be performed 3 times per week throughout the 12 week intervention. To allow for progression, 3 stages of each exercise are provided. Progression is provided by varying the number of repetitions and sets, amount of load, or rate of loading.

   Progression is made when the indicated repetitions and sets are reached for that stage, provided that:

   - Therapist considers that patient can recruit adequate intrinsic muscle contraction without excessive extrinsic contribution, or perform local joint motion at the foot and ankle with optimal control i.e. smooth motion through intended plane of action without unintended overflow into adjacent planes or joints.
   - Participant's rating of subjective effort of each exercise using Modified Borg Scale (see table to right) is 0-5 / 10.
   - Participant's pain response to loading. Pain during exercise <5/10 on a numerical rating scale; no change in pain the following day.

Modified Borg Scale	
0	Nothing at all
1	Very light
2	Light
3	Moderate
4	Somewhat hard
5	Hard
6	
7	Very hard
8	
9	
10	Very very hard

DAILY EXERCISES.

Weeks 1-12 foot and toe movements.
All performed in a seated position. 10 repetitions of each movement.

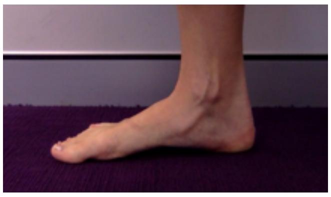

Doming + long toe push

- Gently squeeze the ball of your foot towards your heel
- You should feel a slight lift in your arch
- Gently push your toes into the floor, being sure to keep the toes straight (long)
- Check that:
    - Ankle joint is still
    - Tendon at front of ankle remains relaxed
    - Big toe knuckle stays in contact with ground

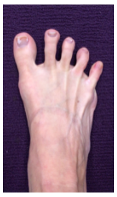

Toe spread

- Lift all toes slightly off the floor
- Spread toes away from each
- Place toes back to the floor
- Check that:
    - Ankle joint is still

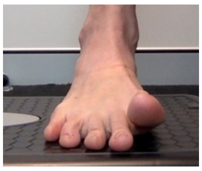

Great toe extension

- Lift the big toe off the floor whilst keeping the other toes in contact with the floor
- Check that:
    - Ankle joint is still
    - Toes are straight (long)

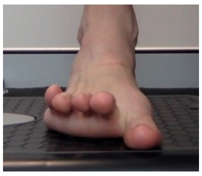

Lesser toe extension

- Lift toes 2-5 off the floor whilst keeping the other toes in contact with the floor
- Check that:
    - Ankle joint is still
    - Toes are straight (long)

Weeks 2-12 <u>continue</u> foot and toe movements <u>and add</u> 2 weight bearing exercises.
5 repetitions of each exercise.

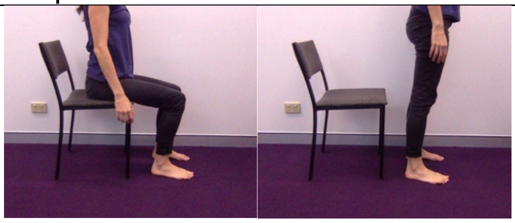

Sit to stand
(with doming + long toe push)

- Sit towards the front of a chair with your feet hip width apart and heels slightly behind your knees.
- Engage good foot position using the doming and long toe push
- Lean forwards at the trunk and stand up
- To reduce difficulty increase seat height and or use of arm support
- To increase difficulty cross arms across chest

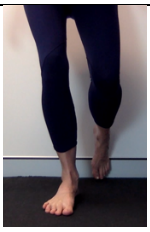

Single leg balance
(with doming + long toe push)

- Use the back of a chair or a wall for fingertip support as required
- Engage good foot position using the doming and long toe push
- Lift one foot just off the ground behind, so that you are standing on one leg and hold for 5-10 seconds (as able)
- Stay ‘tall’ and don’t let your standing side hip sag or slide to the side, keep your knee facing ahead
- To reduce difficulty, increase finger/hand support
- To increase difficulty, reduce finger/hand support

EXERCISES TO PERFORM 3 TIMES A WEEK. Perform 3x/week* throughout 12 week intervention (*One of which will be supervised during physiotherapy session weeks 1, 2, 3, 4, 5, 6, 7, 8, 10).

EXERCISE #1 – DOMING

Stage 1	Stage 2	Stage 3
Two leg squat<br><br>2s down, 2s up<br><br>8-12 reps<br>1-3 sets	Single leg squat<br><br>2s down, 2s up<br><br>8-12 reps<br>1-3 sets	Two leg vertical & horizontal jumps<br><br>30-60bpm<br><br>10 reps<br>1-3 sets
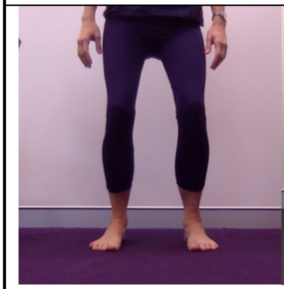 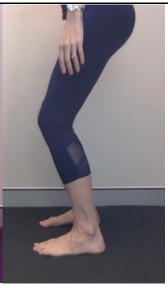<br>• Gently squeeze the ball of your foot towards your heel<br>• You should feel a slight lift in your arch<br>• Be sure to keep your ankle joint still and toes straight (long)<br>• While you hold the doming position, perform a squat as if sitting back towards a chair and then return to standing<br>o You can start with a very small knee bend and progress the depth of the squat as you are able, ensuring good foot position is maintained	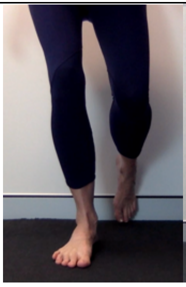 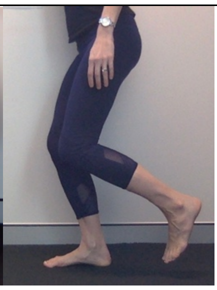<br>• Use the back of a chair or a wall for fingertip support<br>• Gently squeeze the ball of your foot towards your heel<br>• You should feel a slight lift in your arch<br>• Lift one foot just off the ground behind, so that you are standing on one leg<br>• While you hold the doming position, perform a squat as if sitting back towards a chair and back to standing<br>o You can start with a very small knee bend and progress the depth of the squat as you are able, ensuring good foot position is maintained<br>• Don't let your standing side hip sag or slide to the side, keep your knee facing ahead<br>• Increase difficulty by reducing fingertip support	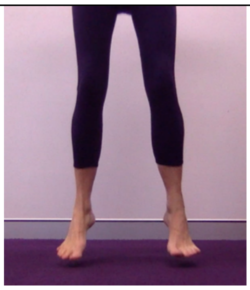<br>• Start with the stage 1 doming exercise (two leg)<br>• While you hold the doming position, perform a small knee bend and jump up (on the spot) or forwards<br>• When you land try to hold the doming position before you relax
**Progress to stage 2 when able to perform**<br>3 sets of 12 reps	**Progress to stage 3 when able to perform**<br>3 sets of 12 reps	**Progress to single leg hop when able to perform**<br>3 sets of 10 reps

EXERCISE #2 – LONG TOE PUSH

**Stage 1**	**Stage 2**	**Stage 3**
Seated<br><br>2s push, 2s release against theraband<br><br>8-12 reps<br>1-3 sets	Standing<br><br>2s push, 2s release against theraband<br><br>8-12 reps<br>1-3 sets	Squat<br><br>2s push, 2s squat down, 2s squat up, 2s release against theraband (progress red to blue)<br><br>8-12 reps<br>1-3 sets
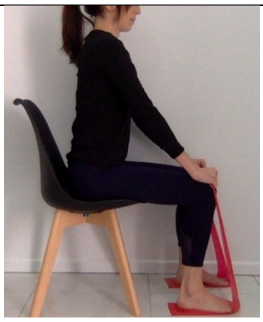<br>• Sit with your feet hip width apart<br>• Place the resistance band flat underneath each foot so that the ball of your foot (knuckle joints) are along the line<br>• Hold the band in each hand at the line number specified by your physiotherapist<br>• Pull upward on the band, allowing the band to extend your toes back, and place your hands on top of your knees<br>• Maintain this position<br>• Keep the toes straight and slowly push them into the band until they reach back down to the floor (2 seconds)<br>• Slowly control your toes extending back to the position you started (2 seconds)	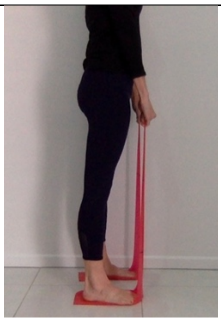<br>• Stand with the resistance band under each foot (ball of foot at line)<br>• Ensure your feet are hip width apart and weight is evenly distributed side to side and front to back<br>• Hold the band in each hand at the line number specified by your physiotherapist<br>• Pull upward on the band until you are standing erect, allowing the band to extend your toes back<br>• Your hands should be resting at the front of your thighs<br>• Maintain this position<br>• Keep the toes straight and slowly push them into the band until they reach back down to the floor (2 seconds)<br>• Slowly control your toes lifting back up to the position you started (2 seconds)	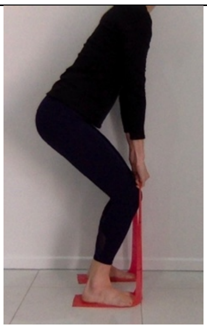<br>• Begin in the same position as Stage 2<br>• Keep the toes straight and slowly push them into the band until they reach back down to the floor (2 seconds)<br>• Hold this position of your foot as you perform a squat, allowing your hands to move down the front of your thighs towards your knee. Perform the squat over 2 seconds down and 2 seconds back up.<br>• Once you return to standing, slowly control your toes lifting back up to the position you started (2 seconds)
**Progress to stage 2 when able to perform**<br>3 sets of 12 reps	**Progress to stage 3 when able to perform**<br>3 sets of 12 reps	**Progress to blue band when able to perform**<br>3 sets of 12 reps

EXERCISE #3 – SEATED HEEL RAISE

**Stage 1**	**Stage 2**	**Stage 3**
Seated<br><br>2s up, 2s down<br><br>8-12 reps<br>1-3 sets	Seated plus resistance<br><br>2s up, 2s down against blue theraband<br><br>8-12 reps<br>1-3 sets	Seated - endurance<br><br>2s up, 2s down against blue theraband<br><br>Reps to fatigue (max 25)<br>3 sets
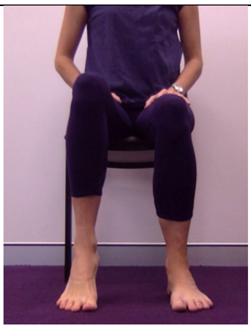<br>• Sit with your feet hip width apart<br>• Engage your foot muscles using the doming and long toe push exercise<br>• Hold this while you lift your heel off the floor over 2 seconds and then lower back down over 2 seconds	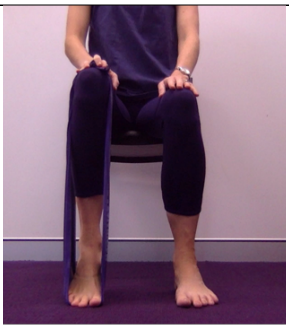<br>• Sit with your feet hip width apart<br>• Place the resistance band under the ball of your foot and over the top of your knee<br>• Engage your foot muscles using the doming and long toe push exercise<br>• Hold this while you lift your heel off the floor over 2 seconds and then lower back down over 2 seconds	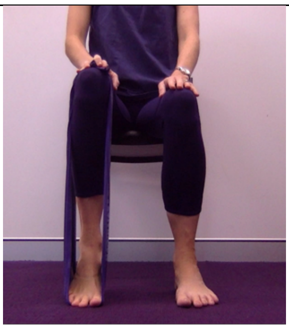<br>• Sit with your feet hip width apart<br>• Place the resistance band under the ball of your foot and over the top of your knee<br>• Engage your foot muscles using the doming and long toe push exercise<br>• Hold this while you lift your heel off the floor over 2 seconds and then lower back down over 2 seconds
**Progress to stage 2 when able to perform**<br>3 sets of 12 reps	**Progress to stage 3 when able to perform**<br>3 sets of 12 reps	**Further progression not required**

EXERCISE #4 – STANDING HEEL RAISE

**Stage 1**	**Stage 2**	**Stage 3**
Two leg<br><br>2s up, 2s down<br><br>8-12 reps<br>1-3 sets	Single leg<br><br>2s up, 2s down<br><br>8-12 reps<br>1-3 sets	Single leg endurance<br><br>2s up, 2s down<br><br>Reps to fatigue (max 25)<br>3 sets
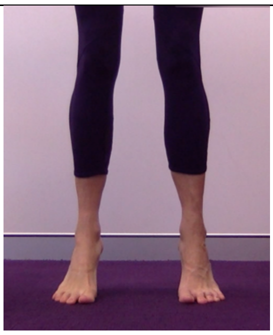<br>• Stand with your feet hip width apart, weight even side to side and front to back<br>• Engage your foot muscles using the doming and long toe push exercise<br>• Hold this while you lift your heels off the floor over 2 seconds and then lower back down over 2 seconds	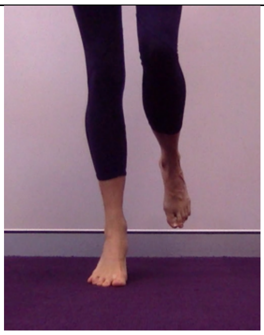<br>• Engage your foot muscles using the doming and long toe push exercise<br>• Stand onto one foot with your weight even from front to back (use fingertip support at a wall or chair back if required)<br>• While you hold the doming position and long toe push, lift your heel off the floor over 2 seconds and then lower back down over 2 seconds<br>• Don't let your standing side hip sag or slide to the side<br>• Increase difficulty by reducing fingertip support	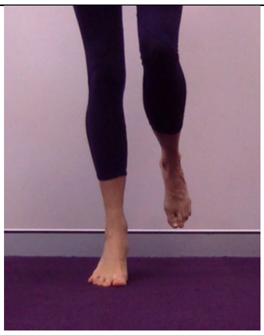<br>• Engage your foot muscles using the doming and long toe push exercise<br>• Stand onto one foot with your weight even from front to back (use fingertip support at a wall of chair back if required)<br>• While you hold the doming position and long toe push, lift your heel off the floor over 2 seconds and then lower back down over 2 seconds<br>• Don't let your standing side hip sag or slide to the side
**Progress to stage 2 when able to perform**<br>3 sets of 12 reps	**Progress to stage 3 when able to perform**<br>3 sets of 12 reps	**Further progression not required**
