# Supplementary material for: Foot exercise plus education versus wait and see for the treatment of plantar heel pain (FEET trial): a protocol for a feasibility study
Source: J Foot Ankle Res. 2020 May 8;13:20. doi: 10.1186/s13047-020-00384-1 (PMC7206811; doi:10.1186/s13047-020-00384-1)

THE FEET TRIAL

The University of Queensland is conducting research to answer important questions about plantar heel pain.

The clinical trial that you are enrolled in will determine which of the most commonly used treatments works best.

For the findings of the study to be worthwhile, you must keep to the treatment which you have been allocated.

If you have any concerns or questions about the trial, please contact Dr Rebecca Mellor at The University of Queensland on:

(07) 3346 7485 or r.mellor@uq.edu.au

The University of Queensland
Australia

CREATE CHANGE

This booklet has been written by physiotherapists at The University of Queensland. The content and images contained within this booklet are subject to copyright, and should not be copied, or distributed without permission.

The University of Queensland
Australia

SIRPH Research Unit, School of Health and Rehabilitation Sciences

Plantar Heel Pain

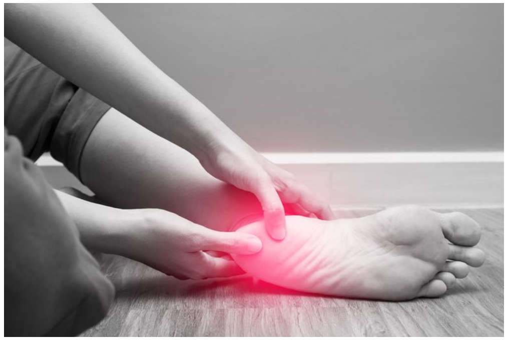

THE FEET TRIAL

Foot **E**xercise and **E**ducation in the **T**reatment of plantar heel pain: A feasibility trial

PLANTAR HEEL PAIN

WHAT IS IT?

The term *plantar heel pain* is a description of symptoms such as:

- Pain located under the sole of the foot at the heel bone
- Pain that is aggravated by weight bearing activities such as prolonged standing, walking or running
- Pain that is particularly acute on first steps in the morning, or following a period of inactivity

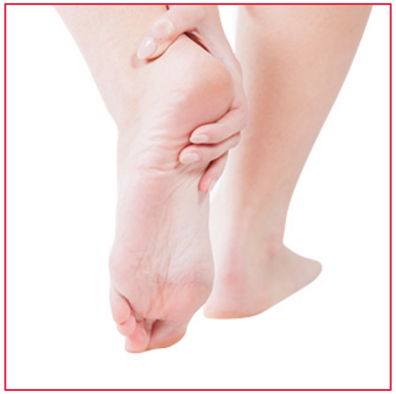

Plantar heel pain is also often referred to as *plantar fasciitis, plantar fasciosis* and *plantar fasciopathy.*

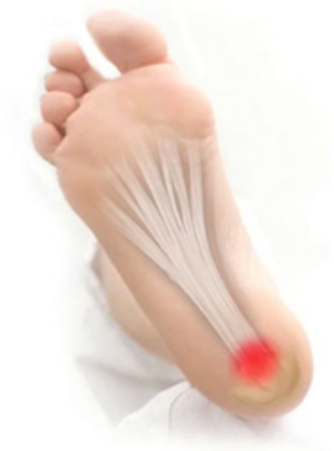

The plantar aponeurosis (or plantar fascia, pictured above) is the most common source of pain, but many structures may be involved. These include the heel bone, the plantar fat pad, nerves, muscles and tendons.

WHY DO I HAVE IT?

Plantar heel pain is the most common cause of foot pain, affecting approximately 10% of the population over the course of a lifetime.

The most common trigger is a change (sudden increase) in weight bearing activity.

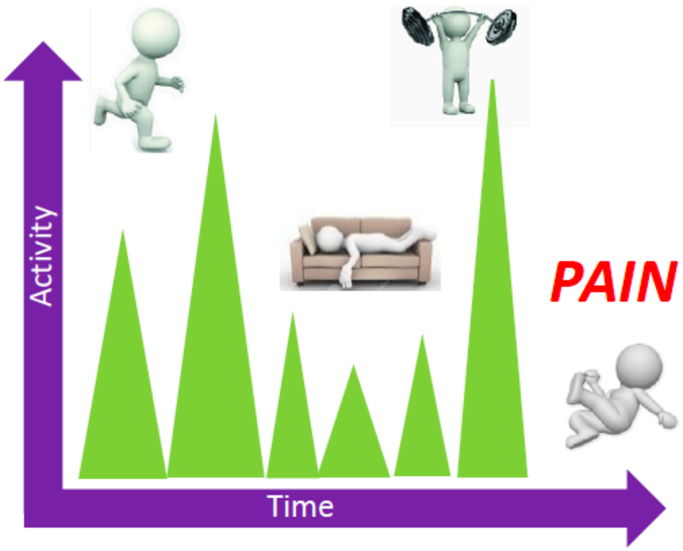

This may be caused by may different factors, for example:

- Increase in weight bearing activities (e.g. change in job, walking holiday)
- Fitness kick / sharp increase in training volume
- Returning too soon following time off, injury or illness
- Weight gain

Sometimes you may not be able to put your finger on a single factor as it might have occurred as a result of an accumulation of a number of small things.

WHAT CAN I DO?

Our approach is a 2 part plan:

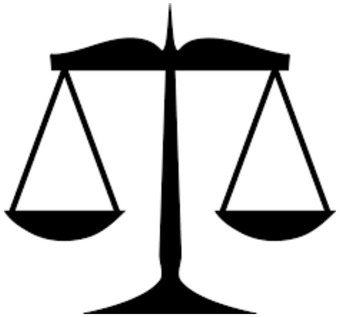

Part 1 is self-management advice that will educate and assist you to monitor and modify weight bearing loads in your occupational and recreational activities, in accordance with your symptoms.

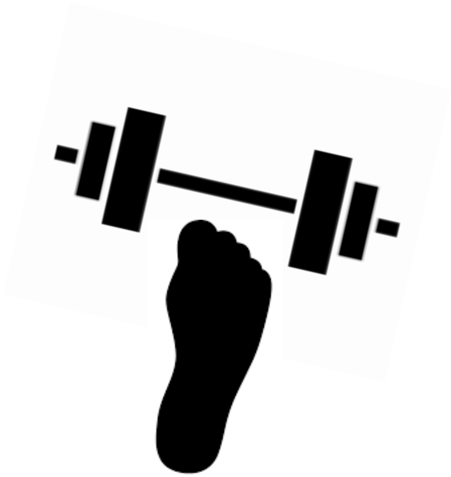

Part 2 is an exercise programme that is specifically designed to gradually strengthen the muscles of your foot.

PART 1: SELF MANAGEMENT ADVICE

MONITOR LOAD & SYMPTOMS

The quickest way to start making a positive difference in your pain is to start modifying weight bearing activities.

Think of all the activities in your occupational, recreational or daily life that involve weight bearing.

As a guide, monitor your pain response:

- During activity
- After activity
- That night
- Next morning

Try to avoid large spikes in activity.

Apply the *Traffic Light* approach:
If your pain stays in the green zone, you can maintain or progress your activity.
If your pain moves into the yellow zone modify your activity until it settles back into the green zone.
If your pain moves into the red zone, stop additional impact activity outside your normal daily activities until it settles back into the yellow zone.

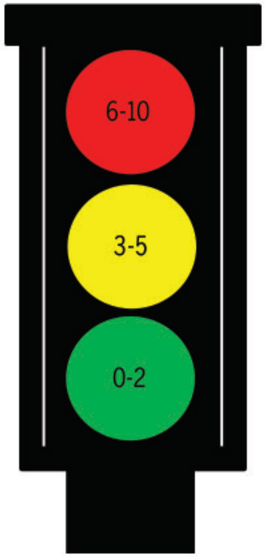

FOOTWEAR AND SURFACES

Try wearing footwear to help to mitigate weight-bearing stresses.

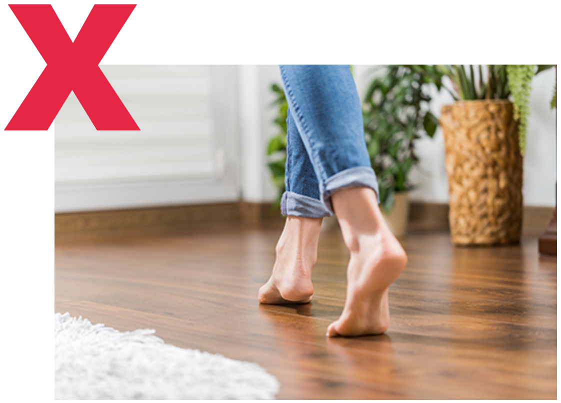

- Avoid barefoot walking, even around the house

- Try a shoe with a cushioned sole e.g. running shoe/sneaker, or sandal/thong with cushioned heel rather than flat

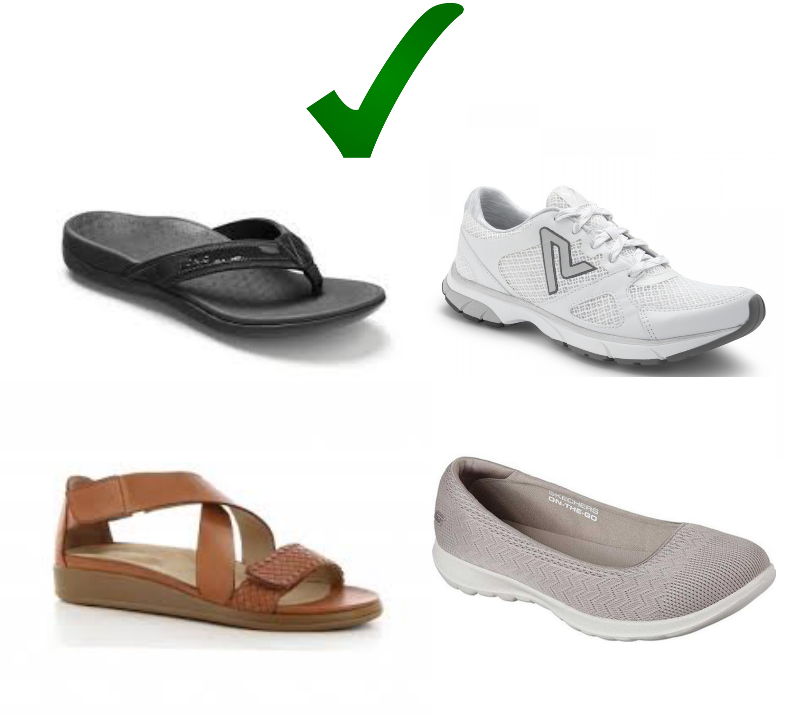

- Try to reduce the time you stand on hard surfaces (e.g. tiles, wooden floors), or mitigate effects by wearing cushioned footwear

POSTURE

If you are standing for a prolonged period:

- Try to distribute your weight evenly between the front to back of your feet

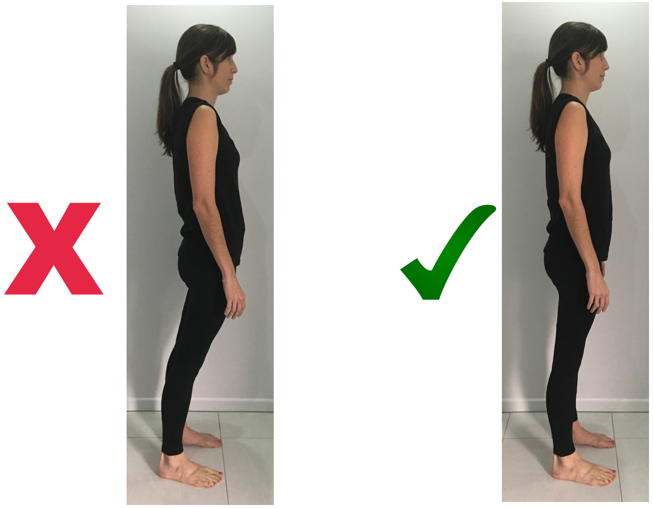

- Try to distribute your weight evenly between each limb

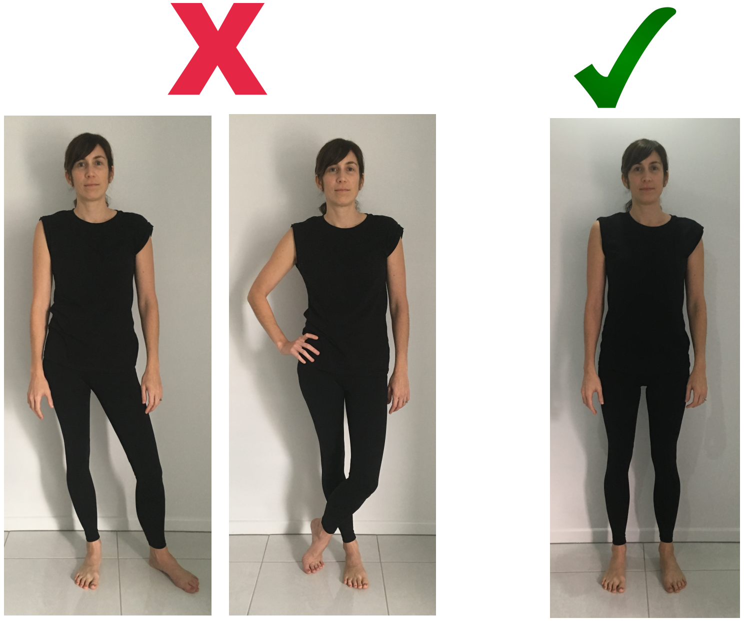

WALKING AND RUNNING

When you are walking or running (if running is an activity you do):

- Take shorter steps

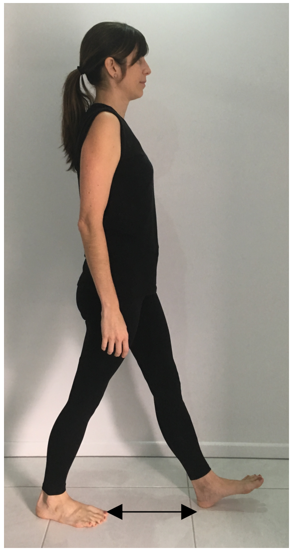

- Walk/run softly by trying to decrease the sound of your footfalls.

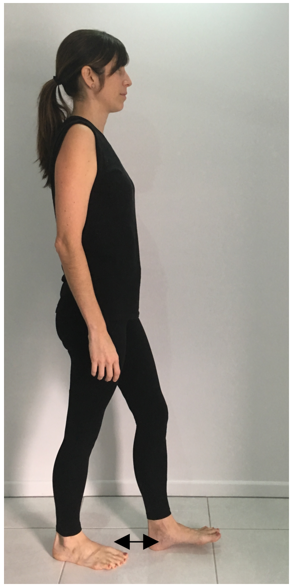

PART 2: EXERCISE

TARGETED FOOT EXERCISE

As part of the study you will attend 8 individual sessions with a physiotherapist.

Your physiotherapist will prescribe a home exercise program that is specifically designed to gradually strengthen the muscles of your foot.

Your physiotherapist will progress exercises as you are able.

It is important that you record the exercises that you complete in the daily logbook that is provided.

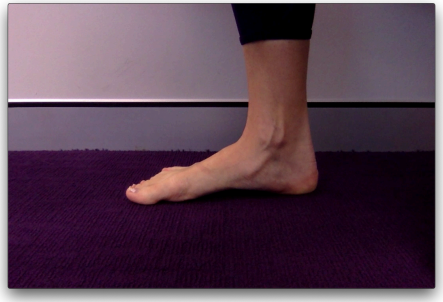

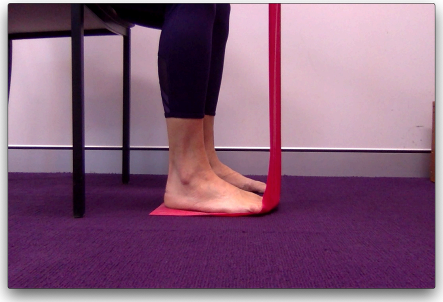

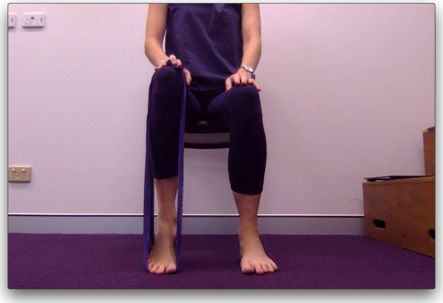

WHAT DO I DO IF I HAVE A FLARE UP OF MY SYMPTOMS?

It is important that you record any flare up of symptoms in your daily logbook, and report it to your physiotherapist.

You can also try some things at home.

- Modify load: Remember the 'traffic light' system. You do not need to stop all activity, but you may find it helpful to reduce the amount of weight-bearing activity/exercise until your symptoms return to a suitable range (yellow or green zone)

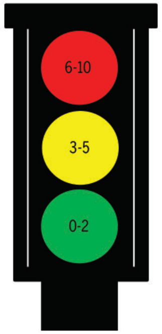

- Apply ice: use a cold pack under your heel, or try rolling your heel over a frozen water bottle

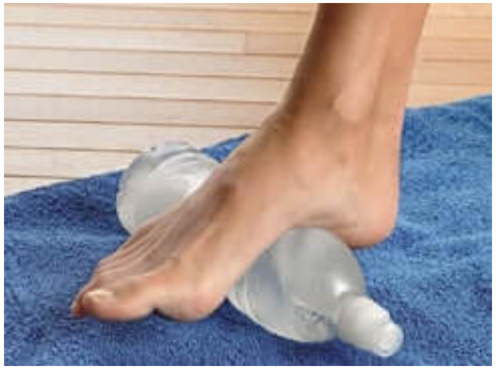

Supplement: Supplementary file 5 — Additional file 5. Foot Exercise and Education group Handout [file 13047_2020_384_MOESM5_ESM.pdf]
