# Supplementary material for: Foot exercise plus education versus wait and see for the treatment of plantar heel pain (FEET trial): a protocol for a feasibility study
Source: J Foot Ankle Res. 2020 May 8;13:20. doi: 10.1186/s13047-020-00384-1 (PMC7206811; doi:10.1186/s13047-020-00384-1)
Supplement: Supplementary file 6 — Additional file 6. Brief Advice group Handout [file 13047_2020_384_MOESM6_ESM.pdf]

WHAT CAN I DO?

For most people, the symptoms of plantar heel pain will resolve within 1 – 2 years.

Rest does not cure plantar heel pain, but activity to the point of feeling pain is also not helpful. Keeping up a level of activity that does not aggravate your pain will be worthwhile.

Start with activities that do not aggravate your pain and as your pain lessens, *gradually* increase your activity levels.

Applying ice to the sole of the foot at the heel and arch can also provide pain relief.

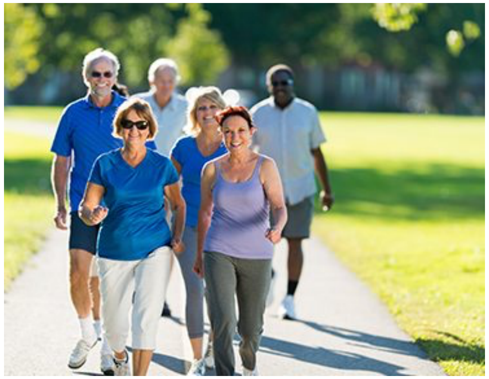

THE FEET TRIAL

The University of Queensland is conducting research to answer important questions about plantar heel pain.

The clinical trial that you are enrolled in will determine which of the most commonly used treatments work best.

For the findings of the study to be worthwhile, you must keep to the treatment which you have been allocated.

If you have any concerns or questions about the trial, please contact Dr Rebecca Mellor at The University of Queensland on:

(07) 3346 7485 or

r.mellor@uq.edu.au

THE UNIVERSITY OF QUEENSLAND
AUSTRALIA

CREATE CHANGE

This booklet has been written by physiotherapists at UQ. The content and images contained within this booklet are subject to copyright, and should not be copied, or distributed without permission.

THE UNIVERSITY OF QUEENSLAND
AUSTRALIA

SIRPH Research Unit, School of Health and Rehabilitation Sciences

Plantar Heel Pain

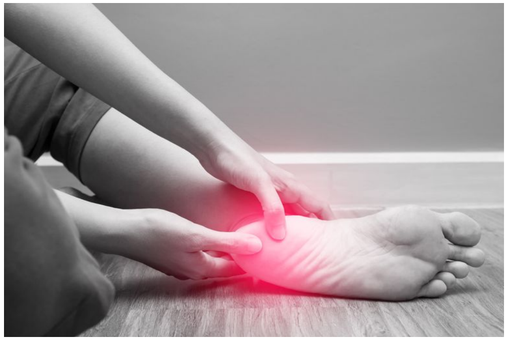

THE FEET TRIAL

Foot **E**xercise and **E**ducation in the **T**reatment of plantar heel pain: A feasibility trial

PLANTAR HEEL PAIN

WHAT IS IT?

The term *plantar heel pain* is a description of symptoms such as:

- Pain located under the sole of the foot at the heel bone
- Pain that is aggravated by weight bearing activities such as prolonged standing, walking or running
- Pain that is particularly acute on first steps in the morning, or following a period of inactivity

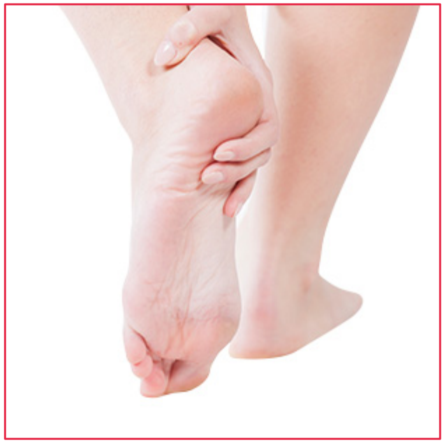

Plantar heel pain is also often referred to as *plantar fasciitis, plantar fasciosis* and *plantar fasciopathy.*

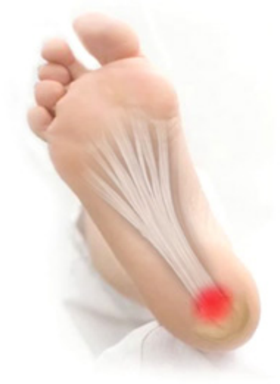

The plantar aponeurosis (or plantar fascia) is the most common source of pain, but many structures may be involved such as the heel bone, the plantar fat pad, nerves, muscles and tendons.

WHY DO I HAVE IT?

Plantar heel pain is the most common cause of foot pain, affecting approximately 10% of the population over the course of a lifetime.

The most common trigger is a change in weight bearing load. This may be caused by many different factors, for example:

- Increase in weight bearing activities (e.g. change in job, walking holiday)
- Fitness kick / sharp increase in training volume
- Returning too soon following time off, injury or illness
- Weight gain

Sometimes you may not be able to put your finger on a single factor as it might have occurred as a result of an accumulation of a number of small things, for example a gradual increase in weight over time and a reduction in general fitness.
