# Supplementary material for: Foot exercise plus education versus wait and see for the treatment of plantar heel pain (FEET trial): a protocol for a feasibility study
Source: J Foot Ankle Res. 2020 May 8;13:20. doi: 10.1186/s13047-020-00384-1 (PMC7206811; doi:10.1186/s13047-020-00384-1)
Supplement: Supplementary file 7 — Additional file 7. Brief Advice group Logbook [file 13047_2020_384_MOESM7_ESM.pdf]

Participant Logbook – *Brief Advice*

Title: **F**oot **E**xercise and **E**ducation in the **T**reatment of plantar heel pain (FEET Trial): A feasibility trial.
Protocol Number: **2019000772**

Principal Investigator: Dr Melinda Smith, School of Health and Rehabilitation Sciences, The University of Queensland
Associate Investigators: Prof Bill Vicenzino, School of Health and Rehabilitation Sciences, The University of Queensland
Dr Natalie Collins, School of Health and Rehabilitation Sciences, The University of Queensland
Dr Rebecca Mellor, School of Health and Rehabilitation Sciences, The University of Queensland
Dr Alison Grimaldi, PhysioTec and School of Health and Rehabilitation Sciences, The University of Queensland

Please record the following information weekly for the 12 weeks of the intervention period, including a rating of your first step pain (firsts steps out of bed/following inactivity):

0 1 2 3 4 5 6 7 8 9 10
No pain — Worst pain imaginable

WEEK	PROBLEMS ENCOUNTERED	ACTION TAKEN	RESPONSE	*FIRST STEP PAIN --/10
1 Date:				
2 Date:				
3 Date:				
4 Date:				
5 Date:				

WEEK	PROBLEMS ENCOUNTERED	ACTION TAKEN	RESPONSE	*FIRST STEP PAIN --/10
6 Date:				
7 Date:				
8 Date:				
9 Date:				
10 Date:				
11 Date:				
12 Date:				
