# Supplementary material for: Foot exercise plus education versus wait and see for the treatment of plantar heel pain (FEET trial): a protocol for a feasibility study
Source: J Foot Ankle Res. 2020 May 8;13:20. doi: 10.1186/s13047-020-00384-1 (PMC7206811; doi:10.1186/s13047-020-00384-1)
Supplement: Supplementary file 8 — Additional file 8. Question guide for semi-structured interviews [file 13047_2020_384_MOESM8_ESM.pdf]

THE UNIVERSITY OF QUEENSLAND AUSTRALIA

Interview Guide

Title: **F**oot **E**xercise and **E**ducation in the **T**reatment of plantar heel pain (FEET Trial): A feasibility trial.

Protocol Number: 2019000772

Principal Investigator: Dr Melinda Smith, School of Health and Rehabilitation Sciences, The University of Queensland

Associate Investigators:
Prof Bill Vicenzino, School of Health and Rehabilitation Sciences, The University of Queensland
Dr Natalie Collins, School of Health and Rehabilitation Sciences, The University of Queensland
Dr Rebecca Mellor, School of Health and Rehabilitation Sciences, The University of Queensland
Dr Alison Grimaldi, PhysioTec and School of Health and Rehabilitation Sciences, The University of Queensland

<u>Record of setting</u>
Interview Location:
Participant ID:
Date:
Number of Attendees:
Name of Physiotherapist/Interviewer:
Name of Transcriber:
Number of Tapes:
Transcription crosschecked:

<u>General introduction & explain the purpose of the interview</u>
Thank you and welcome. My name is Melinda Smith and I am a registered physiotherapist conducting research as part of a research fellowship within the School of Health and Rehabilitation Sciences at The University of Queensland.

I am interested in hearing your perspectives on [self management advice / foot exercise] approach now that you have completed the trial.

Positive, negative or neutral responses are welcome. There are no right or wrong answers, we would like to know as much information as possible about what you think.

<u>Address terms of involvement (i.e. confirm informed consent)</u>
Before we start, I’d like to confirm that you understand that your participation in this study is entirely voluntary and that you may stop the interview at any time. If you do not wish to answer any questions, feel free to decline and we will move on. Please also let me know if you need a break at any time.

Do you have any questions?
Finally, can I confirm that I have your permission to record this interview?

PART 1: Expectations

- Can I confirm which intervention you received [self management advice / foot exercise]?
- How would you describe your thoughts when you were first allocated to this intervention?
- How would you now feel if you were recommended this intervention when visiting a health care professional (e.g. GP, physiotherapist, podiatrist)?
- Would you now recommend this intervention to a friend? If so/if not, why?

PART 2: Description of the intervention

- Can you tell me in your own words what the [self management advice / foot exercise] intervention involved?

PART 3: Adherence to the intervention

- In terms of adhering to the [self management advice / foot exercise] intervention, how did you find it?
- Could you describe what was easy about sticking to the [self management advice / foot exercise] intervention?
- Was there anything you found challenging?
- In your own words, how effective was the [self management advice / foot exercise] intervention for you?

PART 4: Other treatments

- Have you had any other treatments for your heel pain before?
- If so, how did they compare to the [self management advice / foot exercise] approach?
- Did you feel like you had to seek other treatments for your heel pain? If so, why?
- Now that you have experienced the [self management advice / foot exercise], would you seek out other treatment if it was recommended by a physiotherapist or GP? If so, why?

SUMMARY

- Just to summarise, could you tell me in 3 simple bullet points what you felt were the important features of the [self management advice / foot exercise] intervention for you?

End of interview

Is there anything else that you would like to add?
Well, thanks for taking the time to talk with me today. I really appreciate it.

THE UNIVERSITY OF QUEENSLAND AUSTRALIA

Interview Guide - Physiotherapists

Title: Foot **E**xercise and **E**ducation in the **T**reatment of plantar heel pain (FEET Trial): A feasibility trial.

Protocol Number: 2019000772

Principal Investigator: Dr Melinda Smith, School of Health and Rehabilitation Sciences, The University of Queensland

Associate Investigators:
Prof Bill Vicenzino, School of Health and Rehabilitation Sciences, The University of Queensland
Dr Natalie Collins, School of Health and Rehabilitation Sciences, The University of Queensland
Dr Rebecca Mellor, School of Health and Rehabilitation Sciences, The University of Queensland
Dr Alison Grimaldi, PhysioTec and School of Health and Rehabilitation Sciences, The University of Queensland

Record of setting
Interview Location:
Participant ID:
Date:
Number of Attendees:
Name of Physiotherapist/Interviewer:
Name of Transcriber:
Number of Tapes:
Transcription crosschecked:

General introduction & explain the purpose of the interview
Thank you and welcome. My name is Melinda Smith and I am a registered physiotherapist conducting research as part of a research fellowship within the School of Health and Rehabilitation Sciences at The University of Queensland.

I am interested in hearing your perspectives on delivering the [self management advice / foot exercise] approaches now that the trial is completed.

Positive, negative or neutral responses are welcome. There are no right or wrong answers, we would like to know as much information as possible about what you think.

Address terms of involvement (i.e. confirm informed consent)
Before we start, I'd like to confirm that you understand that your participation in this study is entirely voluntary and that you may stop the interview at any time. If you do not wish to answer any questions, feel free to decline and we will move on. Please also let me know if you need a break at any time.

Do you have any questions?
Finally, can I confirm that I have your permission to record this interview?

PART 1: Expectations

- Can I confirm which interventions you delivered [self management advice / foot exercise]?
- Can you describe what your initial expectations of the [self management advice / foot exercise interventions were?
- How would you describe your patients' reactions when they were first allocated to [self management advice / foot exercise] intervention?

PART 2: Description of the intervention

- Can you tell me in your own words what the [self management advice / foot exercise] intervention involved?

PART 3: Adherence to the intervention

- In terms of adhering to the [self management advice / foot exercise] intervention, how did you find it?
- Could you describe what was easy about sticking to the [self management advice / foot exercise] intervention?
- Was there anything you found challenging?
- In your own words, how effective was the [foot exercise] intervention for your patients?
- Would you now recommend [foot exercise] intervention to your patients? If so/if not, why?
- Will you continue to use [foot exercise] intervention in the management of heel pain? If so/if not, why?

PART 4: Other treatments

- Have you delivered any other treatments for heel pain before?
- If so, how did they compare to the [foot exercise] approach?
- Did you feel like you needed/wanted to include other treatments for their heel pain? If so, why?

SUMMARY

- Just to summarise, could you tell me in 3 simple bullet points what you felt were the important features of the [foot exercise] intervention?

End of interview

Is there anything else that you would like to add?
Well, thanks for taking the time to talk with me today. I really appreciate it.
